# Geospatial modeling of environmental and socioeconomic drivers of health outcomes

**DOI:** 10.3389/fpubh.2026.1790559

**Published:** 2026-09-14

**Authors:** Amirhooman Yalali, Hanadi S. Rifai, Omolola E. Adepoju

**Affiliations:** 1 Civil and Environmental Engineering, University of Houston, Houston, TX, United States; 2 Health Systems & Population Science, University of Houston, Houston, TX, United States

**Keywords:** air pollution, chronic disease, geographically weighted regression, Harris County, health disparity, spatial heterogeneity

## Abstract

**Objectives:**

This study aimed to compute the spatially varying associations between seven chronic health conditions—high blood pressure, asthma, chronic obstructive pulmonary disease (COPD), stroke, cancer, diabetes, and frequent mental distress—and key environmental, built-environment, and socioeconomic determinants at the fine, census-tract scale in Harris County, Texas, USA.

**Methods:**

The study uses three methods: Ordinary Least Squares (OLS), Geographically Weighted Regression (GWR), and Multiscale Geographically Weighted Regression (MGWR) to develop association models using 2022 data. The study further validates the developed models using data from 2020 to 2024.

**Results:**

MGWR outperformed both OLS and GWR for every health outcome, increasing the adjusted R^2^ (for example, from 0.57 to 0.75 for blood pressure and from 0.58 to 0.70 for cancer) and reducing the corrected Akaike Information Criterion (AICc) by several hundred points. Poverty and particulate matter (PM_2.5_) were the most consistent predictors, with the strongest positive coefficients concentrated in central, eastern, and southeastern tracts near industrial corridors and in lower-income communities. Different determinants operated at distinct spatial scales, and re-estimating the framework for 2020, 2021, 2023, and 2024 reproduced the 2022 patterns (|ΔR^2^| ≤ 0.09), confirming the stability of the models. These fine-resolution results identify specific high-burden neighborhoods that global models may overlook.

**Conclusion:**

By allowing each determinant to act at its own spatial scale, MGWR produced a transferable framework for identifying vulnerable communities and estimating chronic disease burden where health data are sparse or missing. The fine-resolution, census-tract results can help guide place-based public health interventions toward the eastern and southeastern areas of Harris County where poverty, air pollution, and chronic disease prevalence coincide, supporting more efficient and targeted resource allocation.

## 1 Introduction

Chronic health conditions such as asthma, chronic obstructive pulmonary disease (COPD), stroke, and diabetes are influenced by a complex array of environmental exposures, built-environment characteristics, and socioeconomic factors. Understanding the interplay among the aforementioned variables at a granular spatial level is crucial for effective public health planning and intervention. Chronic diseases are shaped by multiple determinants that often vary significantly across space, even within a single metropolitan area, necessitating analytical frameworks that can capture such spatial heterogeneity.

Environmental pollutants, particularly fine particulate matter (PM_2.5_), have been extensively linked to adverse health outcomes. Studies have demonstrated that elevated PM_2.5_ exposure is associated with increased risks of respiratory and cardiovascular diseases, including asthma, myocardial infarction, and heart failure (1, 2). Ground-level ozone (O_3_), another major pollutant, is known to exacerbate asthma, decrease lung function, and increase the risk of hospitalization due to respiratory complications—especially in areas with high vehicular and industrial emissions (3, 4). The spatial distribution of both PM_2.5_ and ozone concentrations varies significantly, necessitating localized analyses to capture exposure levels and associated health risks accurately. In the present study, exposure was represented by the annual-average PM_2.5_ concentration (μg/m^3^) and the annual average of the daily maximum 8-h ozone concentration (ppb). In Harris County, the dominant local sources of these pollutants—petrochemical and shipping activities along the Houston Ship Channel, together with dense highway traffic—generate steep within-county exposure gradients, motivating the fine-resolution, spatially varying modeling approach adopted here.

Recent large-scale studies have reinforced these aforementioned associations; Anenberg et al. (5) estimated that in 2015, ambient PM_2.5_ and O_3_ were responsible for approximately 5–10 million and 9–23 million asthma-related emergency room visits globally, respectively. Similarly, Di et al. (6) found that in the US Medicare population, a 10 μg/m^3^ increase in PM_2.5_ and a 10 ppb increase in ozone were associated with 7.3 and 1.1% increases in all-cause mortality, respectively. Moreover, research in minority and low-income neighborhoods has shown that higher dietary antioxidant intake can significantly mitigate the adverse cardiovascular impacts of PM_2.5_ exposure, underscoring the complex interactions between environmental pollutants and socioeconomic determinants of health (7). Globally, in the Guangdong-Hong Kong-Macao Greater Bay Area, Zhao et al. (8) reported that long-term exposure to PM_2.5_ and O_3_ in 2019 was linked to 21,113 and 16,286 all-cause deaths, respectively, reflecting a decline in the PM_2.5_ burden but a sharp increase in ozone-related mortality in recent years.

The built environment also plays a pivotal role in shaping health outcomes. Characteristics such as housing age, impervious surface cover, and land-use types influence exposure to environmental stressors and access to public health infrastructure. Older housing has been associated with increased health risks due to factors such as deteriorating infrastructure, poor indoor air quality, and proximity to pollution sources (9, 10). These findings were echoed by Krieger and Higgins (11), who comprehensively documented how poor housing conditions contribute to a wide range of health issues, including respiratory diseases, lead poisoning, injuries, and mental health problems. Urbanization contributes to heat islands, increased stormwater runoff, and pollutant accumulation, particularly in rapidly developing areas such as Harris County, Texas; the study area in this work, where impervious surfaces and land cover change have outpaced infrastructure adaptation (12–15). A long-term study by Hakkenberg et al. (16) showed a significant conversion of green spaces and wetlands to impervious surfaces in Greater Houston between 1997 and 2017, underscoring the spatial dynamics of development and their potential environmental consequences. Furthermore, spatial analysis of urban areas in Japan revealed that certain land cover types—such as urban land, grassland, and water—are positively associated with subjective well-being, whereas bare land correlates negatively with health outcomes (17). The aforementioned studies collectively emphasize the importance of land cover configuration and urban planning in shaping public health trajectories.

Socioeconomic factors further contribute to spatial variability in chronic health outcomes. Higher unemployment rates, poverty levels, and lack of insurance coverage are strongly correlated with increased disease prevalence and reduced access to healthcare services (18, 19). Socioeconomic vulnerabilities are often exacerbated by disproportionate exposure to environmental stressors, creating compounded health burdens for communities. Numerous studies have demonstrated that low socioeconomic status (SES) populations not only face greater exposure to ambient hazards such as fine particulate matter and extreme heat, but also exhibit heightened physiological susceptibility to their effects (20, 21). For example, in urban areas such as New York City, neighborhoods characterized by lower SES have shown significantly higher rates of chronic conditions, such as hypertension and coronary heart disease, underscoring the strong link between social vulnerability and disease burden (22).

Health outcomes in socioeconomically challenged communities become particularly acute during and after environmental disasters. In the aftermath of Hurricane Harvey, such neighborhoods in Harris County experienced longer recovery times, greater pollutant exposure, and more severe mental health stressors (23–25). The extent of flooding itself was found to be inequitably distributed, disproportionately affecting communities with lower socioeconomic status (26, 27).

An extensive body of work has sought to elucidate the relationships among variables that influence community health. Traditional global regression models, such as Ordinary Least Squares (OLS), have been used to associate the environment and infrastructure with health outcomes; however, OLS often fails to account for spatial heterogeneity in relationships among these variables. Geographically Weighted Regression (GWR) addresses this limitation by allowing parameter estimates to vary locally, capturing spatial variations that global models overlook. Building upon this, Multiscale Geographically Weighted Regression (MGWR) enables each explanatory variable to operate at its own spatial scale, providing a more nuanced understanding of spatially varying relationships (28, 29).

Recent studies have leveraged the potential of spatially adaptive models using OLS, GWR, and MGWR. For example, Jia et al. (30) employed MGWR to explore how neighborhood-built-environment characteristics such as residential density and land-use mix influenced walking activity among older adults, revealing that different variables operated over different spatial scales. Similarly, Liu et al. (31) applied GWR to analyze life expectancy trends across China, identifying substantial spatial variability in how socioeconomic and environmental factors affect health outcomes. In urban settings, Wang et al. (32) used GWR to assess how landscape and environmental features influenced residential location choices, emphasizing the heterogeneity of human–environment interactions. Further, Wei et al. (33) emphasized the significance of spatial context in understanding the relationships between built and natural environments and mental health outcomes. Their findings support the premise that spatially varying associations are critical in public health research.

Despite the increasing use of spatial analysis in public health, there remains a knowledge gap in integrating environmental exposures, built-environment features, and socioeconomic variables simultaneously—using multiscale models—to predict a broad spectrum of chronic health outcomes at fine spatial resolutions such as the census-tract level. The majority of the studies to date have focused on isolated health outcomes, single-variable exposures, or larger, jurisdiction-based scales (e.g., counties or states), which may obscure local variability and reduce model accuracy. Moreover, few studies have explored the predictive capabilities of spatial models for reconstructing or estimating health data in areas or during time periods where such data are missing or incomplete.

The current study addresses the aforementioned gaps by integrating multiple health outcomes and determinants into a unified spatial modeling framework that leverages MGWR’s advantages. The study utilizes 2022 census-tract-level data to examine the associations between environmental exposures (PM_2.5_ and ozone), built-environment characteristics (housing age and impervious surfaces), socioeconomic indicators (unemployment, poverty, and lack of insurance), and health outcomes (blood pressure, asthma, COPD, cancer, stroke, diabetes, and mental health disorders). In addition to the primary 2022 analysis, the developed spatial modeling framework was applied to data from 2020 to 2024 to evaluate its reliability and robustness. The study focuses primarily on 2022 data, while the multi-year results are used to validate the applicability of the modeling framework rather than to conduct a rigorous temporal trend analysis. Such an analysis is not entirely feasible because longitudinal data are unavailable for several variables included in the models.

By employing OLS, GWR, and MGWR, this research study aims to the following:

1. Quantify the relationships between multiple environmental and social variables and chronic health outcomes at a relatively fine spatial resolution.
2. Compare the performance of OLS, GWR, and MGWR in capturing spatially varying relationships.
3. Identify regions where certain variables have a stronger or weaker influence on health outcomes.
4. Develop a predictive modeling framework with high explanatory power to estimate missing or incomplete health data.
5. Assess model consistency across multiple years (2020–2024) to enhance confidence in the robustness of the modeled spatial patterns derived from the 2022 data.

The contribution of this study is integrative and methodological rather than introducing a new algorithm. Although the majority of the previous spatial-health analyses have applied GWR or MGWR to a single outcome or a single class of predictors, this study jointly models seven chronic conditions using environmental, built-environment, and socioeconomic predictors within a single MGWR framework at the census-tract scale and benchmarks the results against OLS and GWR. A second element is an explicit multi-year validation: the 2022 models are re-estimated for 2020, 2021, 2023, and 2024 and evaluated with a goodness-of-fit consistency criterion (ΔR^2^ and the corrected Akaike Information Criterion [ΔAICc]), providing a quantitative test of temporal stability that is seldom reported in comparable work. Together, these elements support the use of the framework to estimate chronic disease burden in tracts or years with missing health data. At the same time, the analysis remains associational rather than causal.

## 2 Materials and methods

### 2.1 Study area: Harris County, Texas

Harris County, located in southeastern Texas (Figure 1), is the most populous county in the State and the third most populous in the United States. Home to Houston, the fourth-largest city in the U. S., the county covers 1,778 square miles and is characterized by rapid urbanization, an extensive industrial sector, diverse land cover and land use, and significant environmental challenges (19). Harris County has experienced substantial population growth in recent decades, reaching over 4.8 million residents as of 2023. The county includes multiple cities and municipalities, including Houston, Pasadena, Pearland, Baytown, Katy, Sugar Land, Friendswood, Galena Park, Missouri City, and La Porte. The County is one of the most racially and ethnically diverse regions in the United States, with 45% Hispanic/Latino, 34% White (non-Hispanic), 19% Black/African American, and 7% Asian populations. Additionally, 45% of residents speak a language other than English at home, with Spanish, Chinese, Vietnamese, and Arabic being the most common (19).

**Figure 1:**
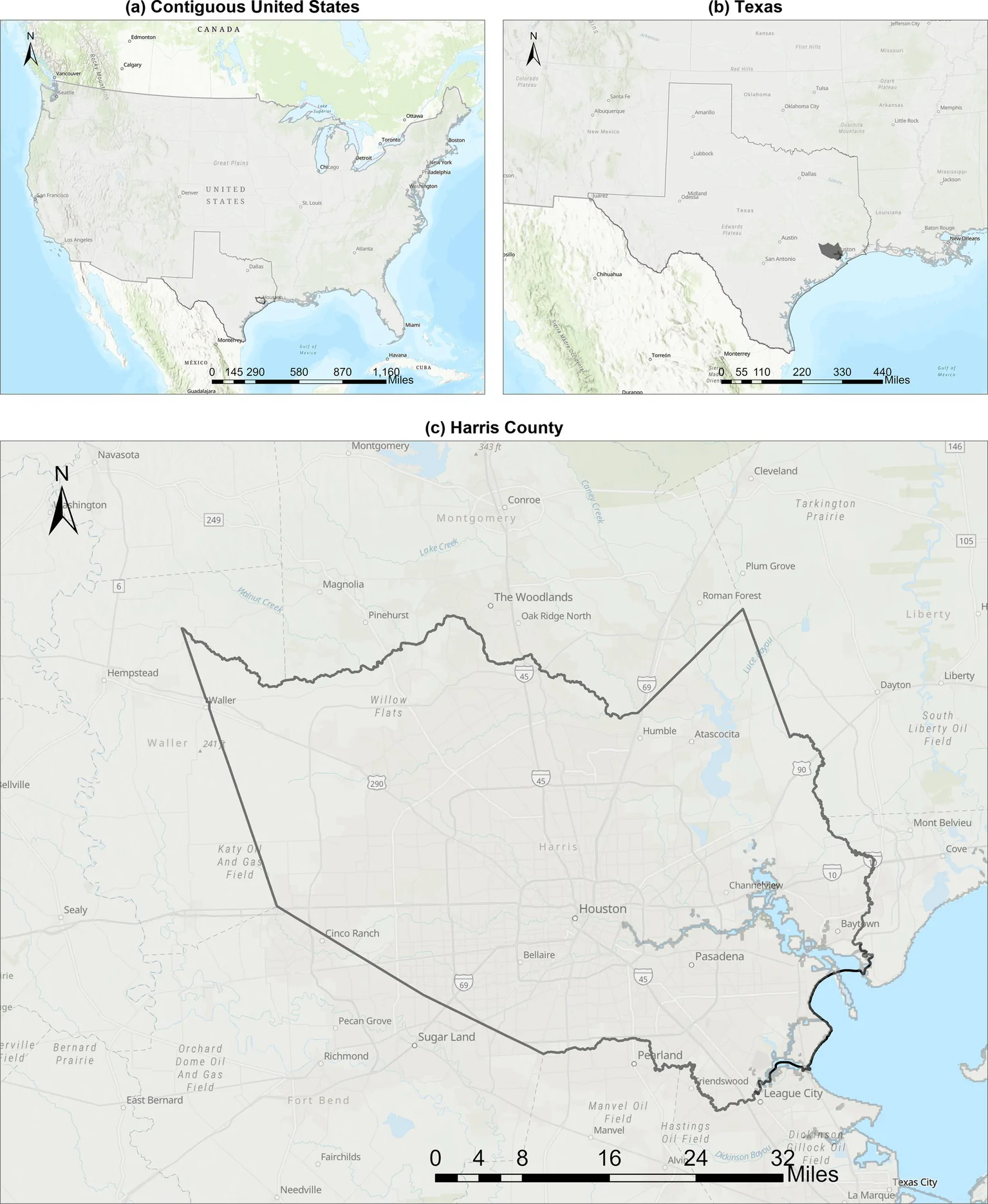
Location of the study area. Harris County, Texas is shown within **(a)** the contiguous United States, **(b)** the State of Texas, and **(c)** at the county scale with major cities. “World Topographic Map” (with World Hillshade). Attribution: “Sources: Esri, TomTom, Garmin, FAO, NOAA, USGS, (c) OpenStreetMap contributors, and the GIS User Community.” License: Esri Master License Agreement (permitted in publications with attribution). Adapted (basemap background with the authors’ data overlaid).

Socioeconomic factors play a significant role in shaping the health of Harris County residents as noted above. As of 2023, the median household income is $70,789, which is lower than both Texas and US averages. One in seven residents in Harris County lives below the poverty line, with high-poverty ZIP codes that include 77,060, 77,037, 77,093, 77,029, and 77,013. Additionally, one in four adults lacks health insurance, and one in five residents cannot afford medical care, further exacerbating health outcomes (19). Harris County has undergone rapid land-use changes over the past three decades, with significant increases in impervious surfaces due to urban expansion. The loss of forests and wetlands has contributed to higher flood risks (34). The County’s land use consists of urban, residential, commercial, and heavy industrial areas. Studies indicate that developed land use has expanded significantly, while forest coverage has declined over time.

Air quality remains a major concern in Harris County, which consistently receives failing grades for ozone (O_3_) and fine particulate matter (PM_2.5_) pollution. Major contributors to these pollutants include industrial emissions from the Houston Ship Channel industries, vehicular emissions from highways and airports, and meteorological factors such as extreme heat events that intensify photochemical reactions (35). A long-term study by Soleimanian et al. (36) found that although ozone levels across the Houston-Galveston-Brazoria region have generally declined, concentrations remain above national standards in many areas, particularly near industrial zones. Their findings highlight the dominant influence of petrochemical emissions and solvent-based industries on surface ozone formation, with some monitoring sites near the Ship Channel exhibiting the highest concentrations.

Similarly, PM_2.5_ pollution poses a substantial health burden for Harris County residents. A geospatial health impact analysis by Bryan and Landrigan (37) estimated that in 2016, Harris County alone experienced over 1,300 premature deaths attributable to PM_2.5_ exposure, along with significant increases in cases of stroke, asthma, Alzheimer’s, and low birthweight cases. The study found that 98.9% of Texas residents live in areas where PM_2.5_ levels exceeded the World Health Organization’s recommended annual average of 5 μg/m^3^, with Harris County among the areas with the highest concentrations in the state. Exposure to these pollutants has been consistently linked to elevated risks of cardiovascular diseases, asthma, lung cancer, and neurological conditions, as mentioned previously.

In addition to air quality challenges, Harris County is highly susceptible to flooding, with 22 major watersheds and flat terrain leading to slow drainage and extreme flood risks (34). Hurricane Harvey, which struck in 2017, was one of the most devastating storms in Houston’s history, causing approximately $125 billion in economic damages. The storm led to extreme flooding, with over 204,000 homes and buildings damaged (34). Thousands of residents were displaced, particularly in low-income areas. Floodwaters overwhelmed sewage treatment plants, leading to widespread water contamination. Additionally, at least four Superfund sites in Harris County were flooded, with some reports indicating up to 13 sites in the broader region (24, 38).

Chronic diseases are prevalent among Harris County residents. As of 2023, 7% of residents have asthma, while obesity affects 34.3% of the population (19). High blood pressure is reported in approximately 28–32.4% of adults. Diabetes prevalence is 9.7% among adults aged 20 + in Harris County but can reach as high as 22.5% in certain high-risk communities. Stroke prevalence varies between 5.4 and 7%, depending on the population studied (39, 40).

Natural disasters, particularly Hurricane Harvey, have also exacerbated mental health conditions. Reports indicate that 21% of residents showed probable depression, while 17% reported symptoms of anxiety. Women and low-income residents reported the highest levels of stress following Hurricane Harvey (23). High-poverty neighborhoods are disproportionately exposed to extreme weather events, while limited healthcare access contributes to worsening chronic disease outcomes.

Harris County is an ideal location for a pilot study to assess the relationships among environmental quality, land use, climate vulnerability, and public health outcomes. Its rapidly growing population and extensive industrial sector contribute to significant air pollution and land use changes, exacerbating environmental health impacts. The County’s susceptibility to extreme weather events, including hurricanes, flooding, and heat waves, further intensifies risks to vulnerable populations. Additionally, the presence of diverse racial, ethnic, and socioeconomic communities provides an opportunity to examine the variabilities in exposure and health outcomes at the community scale. The intersection of these factors makes Harris County a useful case study for understanding how environmental and public health challenges manifest in urbanized and industrialized regions. The findings from this study can inform decision-makers in other urban areas in the United States and around the globe.

### 2.2 Data sources

The variables used in the study span four major categories: health outcomes, air quality, built-environment, and socioeconomic indicators. Table 1 summarizes the data sources and spatial resolution for each variable.

- Impervious surface data: Built-environment intensity was represented by percent impervious surface data derived from the Multi-Resolution Land Characteristics Consortium (41). The dataset provides 30-m resolution raster values representing the percentage of impervious cover (ranging from 0 to 100%) for each grid cell across the United States. To align with the census-tract boundaries used in the study, the impervious surface raster was imported into ArcGIS Pro, and the Zonal Statistics as Table tool was used to calculate the mean imperviousness for each tract. The process yielded a single percentage value per tract, representing the average impervious surface cover, which was used as a proxy for urban development, land-cover modification, and potential environmental exposure related to stormwater, heat, and infrastructure.
- Air quality data: Air quality data for PM_2.5_ and ozone (O_3_) were obtained from the US Environmental Protection Agency’s (EPA’s) Remote Sensing Information Gateway (RSIG, EPA 2025), specifically the downloadable datasets derived from the Hierarchical Bayesian Model (HBM) framework. Annual average pollutant surfaces were created by fusing ground-based air monitoring, satellite data, and model estimates, producing continuous surfaces at a 12-km resolution across the United States. Using ArcGIS, the pollutant concentration rasters were aggregated at the census-tract level via the Zonal Statistics tool, generating tract-level average values for PM_2.5_ and ozone. This enabled integration of air-quality measures with the other study variables at a consistent spatial resolution.
- Other Variables: Beyond imperviousness and air quality, several other variables and their values were assembled for the study. These include:

i Health outcomes (asthma, COPD, diabetes, stroke, cancer, mental health disorders, and high blood pressure) were sourced from the US Centers for Disease Control and Prevention (CDC) Population Level Analysis and Community Estimates (PLACES) Project, which provides small-area estimates using Behavioral Risk Factor Surveillance System (BRFSS) data and model-based inference.
ii Housing age was obtained from the American Community Survey (ACS) 5-Year Estimates, using the “median year built” of housing units.
iii Socioeconomic indicators, including unemployment, poverty, and lack of insurance, were also derived from ACS and linked by census-tract identifiers.

**Table 1:** Study variables and their sources.

Variable category	Variable name	Source/unit
Health outcomes	Asthma, COPD, stroke, cancer, diabetes, mental health, high blood pressure	CDC PLACES Project, %
Air quality	Annual average PM_2.5_ concentration	EPA RSIG and HBM model
	Annual average O_3_ concentration	
Built environment	Houses built pre-1980	US Census Bureau—ACS 5-year estimates, %
	Average impervious surface per tract	Multi-Resolution Land Characteristics Consortium, %
Socioeconomic	Unemployment	ACS 5-year estimates, %
	Poverty	
	Lack of health insurance	

### 2.3 Modeling and geostatistical methods

#### 2.3.1 Ordinary Least Squares (OLS) regression

The OLS framework serves as a baseline model for identifying general associations and spatial trends without accounting for local variation. The model takes the form given in Equation 1:

$$\begin{array}{l} Y_{i}=\beta_{0}+\alpha \beta_{1}\;{\mathrm{PM}}_{2.5,i}+\alpha \beta_{2}\;{\text{Ozone}}_{i}+\alpha \beta_{3}\;{\text{Impervious}}_{i}\\ +\alpha \beta_{4}\;{\text{HousingAge}}_{i}+\beta_{5}\;{\text{Poverty}}_{i}+\beta_{6}\;{\text{Unemployment}}_{i}\\ +\beta_{7}\;{\text{Uninsured}}_{i}+\varepsilon_{i} \end{array}$$ (1)

where Y_i_ represents a chronic health outcome (e.g., asthma, diabetes, and stroke) in census-tract i, and the β coefficients represent the relative global (non-spatially variable) influence of each independent variable. In the initial stage of analysis, the OLS regression model was applied to assess the relationships between health outcomes and their potential predictors in Harris County. To assess multicollinearity among the independent variables in the OLS regression models, Variance Inflation Factor (VIF) values were calculated. VIF indicates how much the variance of an estimated regression coefficient increases due to collinearity among predictors. VIF values greater than 5 are generally considered indicative of moderate multicollinearity, while values above 10 may signal serious multicollinearity issues. In this study, all VIF values were below 5, suggesting that multicollinearity was not a major concern and that the estimated coefficients were reasonably stable and interpretable (42). A detailed listing of the dependent and independent variables in the OLS analysis is shown in Table 2.

**Table 2:** Description of dependent and independent variables.

Role	Variable
Dependent variables	High blood pressure among adults ≥18 years (%)
Dependent variables	Chronic obstructive pulmonary disease among adults ≥18 years (%)
Dependent variables	All cancer (excluding skin cancer) among adults ≥18 years (%)
Dependent variables	Stroke among adults ≥18 years (%)
Dependent variables	Diagnosed diabetes among adults ≥18 years (%)
Dependent variables	Frequent mental distress among adults ≥18 years (%)
Dependent variables	Current asthma among adults ≥18 years (%)
Independent variables	Annual average of daily 24-h average PM₂.₅ concentration (μg/m^3^)
Independent variables	Annual average of daily maximum 8-h O_3_ concentration (ppb)
Independent variables	Fraction impervious surface (%)
Independent variables	Houses built pre-1980 (%)
Independent variables	Lack of health Insurance (%)
Independent variables	Unemployment (%)
Independent variables	Poverty below 200 (%)

#### 2.3.2 GWR and MGWR

OLS regression, described above, often masks local dynamics by producing global averages (43, 44). As a result, important spatial variation in relationships may remain hidden (45). Geospatial Weighted Regression (GWR) addresses this by calibrating a separate regression equation at each observation point, enabling an evaluation of how relationships change over space (46–51).

Geographically Weighted Regression (GWR) is a spatial regression technique developed to capture non-stationary relationships between dependent and independent variables across geographic space. Unlike OLS, GWR accounts for spatial variation by estimating local parameters at each location. The method is particularly well suited for modeling health, environmental, or social phenomena that exhibit regional variability.

The general form of a GWR model is given by (47, 52).

$$Y_{i}=\beta_{0}(i)+\sum_{k=1}^{p}\alpha \beta_{k}(i)X_{\mathrm{ik}}+\varepsilon_{i},\;\;i=1,\ldots ,n$$ (2)

In Equation 2, $\beta_{k}$ (i) varies by location i, thereby capturing local variations in the relationship between the predictor X_ik_ and the outcome Y_i_.

The GWR estimator is given by Equation 3:

$$\hat{\beta }(i)={\left(X^{T}W(i)X\right)}^{-1}X^{T}W(i)Y$$ (3)

Here, W(i) is a spatial weighting matrix specific to location i, usually determined by a kernel function that assigns higher weights to observations closer to i (52–54).

The core principle of GWR is that the method uses data from neighboring locations, giving greater weight to observations that are geographically closer to the target location where the local regression is estimated. This proximity-based weighting ensures that the model is calibrated specifically to each location i, capturing local dynamics while minimizing the influence of distant data points. The logic behind this approach is that spatially closer features are more likely to share similar relationships and characteristics (28). The spatial weights are determined using a kernel function, and the bandwidth of this kernel—i.e., how far influence extends—is optimized during the model calibration process.

To evaluate the performance of the GWR model, three diagnostic tools were used:

- R^2^ (Coefficient of Determination): This metric assesses the model’s goodness of fit, with values ranging from 0 (poor fit) to 1 (perfect fit). In the context of GWR, R^2^ measures the proportion of variance in the dependent variable (e.g., asthma and diabetes) explained by the spatially varying regression model (47).
- Akaike Information Criterion Corrected (AICc). The AICc is calculated as shown in Equation 4:

$$\mathrm{AI}C_{c}=2n\cdot \ln(\hat{\sigma })+n\cdot \ln(2\pi )+\frac{2n(n+\backslash \mathrm{tr}(S))}{n-2-\backslash \mathrm{tr}(S)}$$ (4)

where n is the sample size, $\hat{\sigma }$ is the estimated standard deviation (SD) of residuals, and tr(S) is the trace of the hat matrix S, representing model flexibility. Bandwidth selection (how far the variable influence extends) was performed by minimizing the AICc, which balances model fit and complexity.

- Spatial Autocorrelation (Moran’s I). Moran’s I is calculated using Equation 5 (55):

$$I=\frac{n}{S_{0}}\cdot \frac{\sum_{i=1}^{n}\sum_{j=1}^{n}W_{\mathrm{ij}}(x_{i}-\bar{x})(x_{j}-\bar{x})}{\sum_{i=1}^{n}{\left(x_{i}-\bar{x}\right)}^{2}}$$ (5)

where

- n is the number of spatial units (e.g., census tracts),
- x_i_ and x_j_ are the values of the variable at locations i and j,
-
$\bar{x}$ is the mean of all values,
- W_ij_ is the spatial weight between location i and j, where adjacent tracts have weights of 1 and non-adjacent tracts have weights of 0, and
- S_0_ is the sum of all spatial weights.

Moran’s I values range from −1 to +1:

- A value near +1 indicates positive spatial autocorrelation (i.e., similar values cluster together),
- A value near −1 indicates negative spatial autocorrelation (i.e., dissimilar values cluster),
- A value close to 0 suggests no spatial autocorrelation, meaning the spatial pattern is likely random (56).

Moran’s I enables an evaluation of the presence of spatial autocorrelation in model residuals (57). Index values compute the degree to which similar or dissimilar values of a variable are spatially grouped, offering insight into whether observed spatial patterns are random or systematically structured. Significant spatial autocorrelation in the residuals may suggest that an important explanatory variable is missing or that an unmodeled spatial process is influencing the results. Addressing this is critical, both before and after GWR calibration, as GWR is especially useful when residuals from a global model (such as OLS) exhibit spatial dependence or when coefficients are suspected to vary across space.

To test the statistical significance of Moran’s I, the following hypotheses were evaluated:

- H₀: There is no spatial autocorrelation (Moran’s I ≈ 0),
- H₁: There is significant spatial autocorrelation (positive or negative).

The test was used both before applying GWR/MGWR—to justify the need for spatial models—and after model calibration—to assess whether the regression captured spatial structure. While GWR effectively accounts for spatial heterogeneity in relationships, it assumes that all explanatory variables vary across space at the same spatial scale. This uniform bandwidth assumption can oversimplify the reality that different processes—such as air pollution exposure versus socioeconomic vulnerability—may operate over vastly different spatial extents.

Multiscale Geographically Weighted Regression (MGWR) addresses the aforementioned limitation of GWR by enabling variable-specific bandwidths, allowing each covariate’s spatial influence to be optimized independently (29).

The MGWR model is formally expressed as shown in Equation 6:

$$y_{i}=\beta_{0}(bw_{0},u_{i},v_{i})+\sum_{j=1}^{p}\beta_{j}(bw_{j},u_{i},v_{i})\;x_{\mathrm{ij}}+\varepsilon_{i}$$ (6)

Here, bw_j_ represents the bandwidth used to calibrate the spatial influence of the jth explanatory variable, and (u_i_,v_i_) are the coordinates for observation i. By allowing distinct bandwidths for each predictor, MGWR can capture, for example, that PM_2.5_ exposure may affect health outcomes at a broader regional scale, whereas variables such as housing age or poverty may exert more localized effects.

In the current study, separate MGWR models were developed for each chronic health condition; this was undertaken in order to identify spatially varying relationships among the predictors (PM_2.5_, ozone, impervious surface, housing age, poverty, unemployment, and lack of insurance) and health outcomes. As mentioned above, the main analysis focuses on 2022 data; however, the same modeling framework was applied to data from 2020 through 2024 to evaluate the stability of the developed models and the reliability of the resulting spatial patterns over time. The 2020 through 2024 model runs were not used to examine temporal trends, but rather to confirm that the associations observed in 2022 were robust and consistent across multiple years, strengthening the validity of the findings.

The calibration of MGWR was performed using a back-fitting algorithm, which begins with initial parameter estimates derived from a standard GWR model. During each iteration, the algorithm updates local coefficients and recalculates optimal bandwidths for each variable until convergence is reached. During calibration, convergence was achieved when the difference between successive parameter estimates fell below a threshold of 1 × 10^−5^, following the procedures outlined in Fotheringham et al. (29) and Oshan et al. (58). Selecting an appropriate bandwidth is a critical step. A bandwidth that is too large reduces the model’s ability to detect spatial non-stationarity—essentially reverting the model to a global form similar to OLS. Conversely, a bandwidth that is too small can produce highly variable and unstable local estimates. MGWR enables a balance between these extremes by optimizing each covariate’s bandwidth individually, making it well-suited to studying phenomena influenced by diverse processes across varying geographic scales (28), such as those in this study. As both GWR and MGWR generate local parameter estimates for each spatial unit, the results from the two methods represent distributions of local coefficients rather than single global estimates. To summarize the generated distributions, the mean, median, and standard deviation of local coefficients are reported in the manuscript. The mean and median capture central tendencies across space, while the standard deviation captures spatial variability, enabling clearer interpretation of non-stationarity and comparison with global OLS estimates. All OLS, GWR, and MGWR models were calibrated using the open-source MGWR software developed by Oshan et al. (58) and Fotheringham et al. (29) (the MGWR Python package), and all spatial data preparation, zonal statistics, and mapping were performed in ArcGIS Pro (Esri). Variance Inflation Factors and descriptive statistics were computed in Python. Prior to model calibration, all dependent and independent variables were standardized to z-scores, so that the resulting local coefficient estimates could be directly comparable in magnitude across predictors. Census tracts with missing values for any modeled variable (indicated in pink in the figures) were excluded from the corresponding regressions.

## 3 Results

### 3.1 Spatial variability of Harris County data

Figure 2 provides a spatial visualization of the distribution of select environmental, built-environment, socioeconomic, and health-related variables across Harris County census tracts. The figure shows that the values for each variable are significantly heterogeneous, with no apparent trends discernible by visual inspection. Some general observations can be made; for example, diseases such as asthma, blood pressure, stroke, and diabetes exhibit their highest rates in overlapping communities. Environmental variables such as PM_2.5_ and ozone exhibit distinct geospatial distributions, suggesting that their sources and causes are likely different and that they affect health differently. Poverty, mental health, housing age, and lack of insurance are prevalent in very similar spatial patterns. The data in Figure 2 illustrate the need for the modeling that was undertaken in this study; visual observations are very useful but cannot fully inform decision-making or developing impactful and actionable interventions to improve health outcomes. Additionally, understanding the collective impact of various variables on health cannot be achieved solely through visual observation.

**Figure 2:**
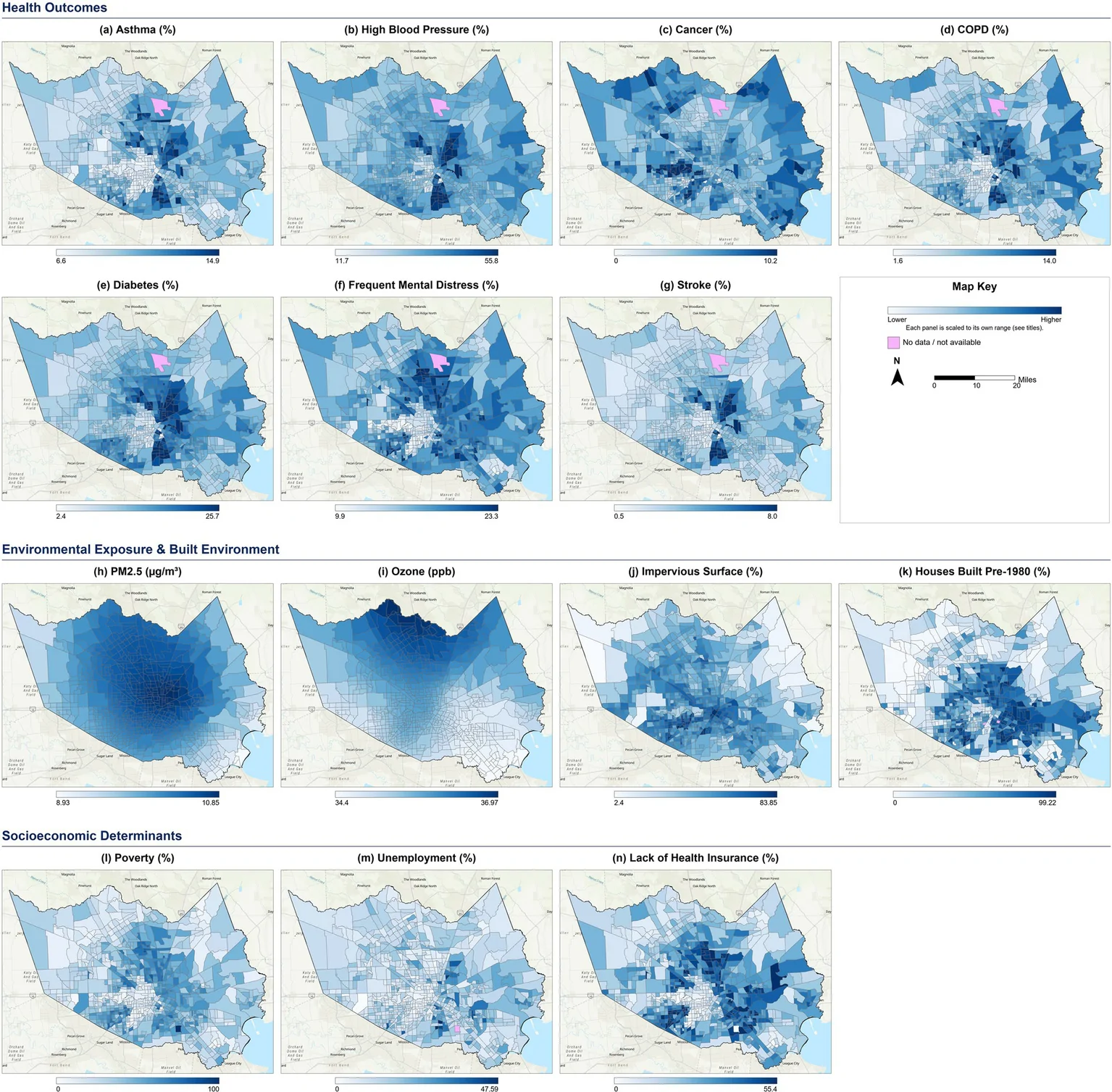
Visualization of study variables (in %) in Harris County at the census-tract scale (2022 data). Pink areas indicate census tracts with missing or unavailable data. **(a)** asthma, **(b)** high blood pressure, **(c)** cancer, **(d)** COPD, **(e)** diabetes, **(f)** frequent mental distress, **(g)** stroke, **(h)** PM2.5, **(i)** ozone, **(j)** impervious surface, **(k)** houses built pre-1980, **(l)** poverty, **(m)** unemployment, **(n)** lack of health insurance; plus a map key.

### 3.2 OLS and spatial autocorrelation

Table 3 summarizes the OLS regression results for each health outcome, including coefficient estimates, *p*-values, VIF, adjusted R^2^, and AICc for 2022 (Supplementary Tables S1–S4 present the results for the other modeled years). Overall, socioeconomic variables such as poverty and the uninsured rate consistently showed strong and statistically significant associations with chronic health outcomes.

**Table 3:** Statistical modeling results using Ordinary Least Squares regression (2022 data).

Outcome	Statistic	Housing age	Poverty rate	Unemployment rate	Uninsured rate	Impervious surface	Ozone	PM2.5
Blood pressure	Coefficient (*p*-value)	0.069*** (<0.001)	0.222*** (<0.001)	0.424*** (<0.001)	−0.251*** (<0.001)	−0.117*** (<0.001)	−0.197 (0.428)	−0.209 (0.712)
	VIF	1.798	3.911	1.203	3.612	1.417	4.269	3.407
	Adjusted R^2^	0.56						
	AICc	4,458						
Cancer	Coefficient (*p*-value)	0.026*** (<0.001)	−0.018*** (<0.001)	0.012 (0.131)	−0.062*** (<0.001)	−0.017*** (<0.001)	0.118* (0.031)	−0.087 (0.409)
	VIF	1.798	3.911	1.203	3.612	1.417	4.269	3.407
	Adjusted R^2^	0.57						
	AICc	2061						
Asthma	Coefficient (*p*-value)	0.061*** (<0.001)	0.083*** (<0.001)	0.012 (0.131)	−0.030*** (<0.001)	−0.013*** (<0.001)	0.066 (0.078)	0.100 (0.222)
	VIF	1.798	3.911	1.203	3.612	1.417	4.269	3.407
	Adjusted R^2^	0.74						
	AICc	1,534						
COPD	Coefficient (*p*-value)	0.023*** (<0.001)	0.074*** (<0.001)	0.088*** (<0.001)	−0.043*** (<0.001)	−0.028*** (<0.001)	0.331*** (<0.001)	0.295* (0.020)
	VIF	1.798	3.911	1.203	3.612	1.417	4.269	3.407
	Adjusted R^2^	0.73						
	AICc	2,180						
Diabetes	Coefficient (*p*-value)	0.034*** (<0.001)	0.146*** (<0.001)	0.205*** (<0.001)	−0.054*** (<0.001)	−0.063*** (<0.001)	−0.009135	0.340 (0.245)
	VIF	1.798	3.911	1.203	3.612	1.417	4.269	3.407
	Adjusted R^2^	0.74						
	AICc	3,391						
Mental health	Coefficient (*p*-value)	−0.007*** (<0.001)	0.097*** (<0.001)	0.061*** (<0.001)	0.045*** (<0.001)	−0.004 (0.120)	0.503*** (<0.001)	0.621*** (<0.001)
	VIF	1.798	3.911	1.203	3.612	1.417	4.269	3.407
	Adjusted R^2^	0.86						
	AICc	2,146						
Stroke	Coefficient (*p*-value)	0.011*** (<0.001)	0.048*** (<0.001)	0.084*** (<0.001)	−0.041*** (<0.001)	−0.020*** (<0.001)	−0.076 (0.072)	0.014 (0.882)
	VIF	1.798	3.911	1.203	3.612	1.417	4.269	3.407
	Adjusted R^2^	0.67						
	AICc	1,697						

*indicates statistical significance at the 5% level (*p* < 0.05), ** at the 1% level (*p* < 0.01), and *** at the 0.1% level (*p* < 0.001).

The mental health model demonstrated the highest explanatory power (adjusted R^2^ = 0.866), with strong positive associations for ozone (β = 0.503, *p* < 0.001) and PM₂.₅ (β = 0.621, *p* < 0.001), as well as with poverty and unemployment. The asthma model (R^2^ = 0.748) also revealed strong effects of poverty and housing age, and a negative effect of the uninsured rate.

The COPD (R^2^ = 0.737) and diabetes (R^2^ = 0.741) models showed significant contributions from air pollutants and socioeconomic factors, particularly ozone, PM_2.5_, poverty, and unemployment. In contrast, the blood pressure (R^2^ = 0.567) and cancer (R^2^ = 0.576) models had moderate explanatory power. Blood pressure was mainly influenced by unemployment (β = 0.424, *p* < 0.001) and poverty, while cancer showed significant associations with housing age, uninsured rate, and ozone. Across all models, impervious surface had a consistently relatively small but statistically significant negative relationship with health outcomes.

To assess the presence of spatial dependence in the residuals of the OLS models, Moran’s I tests were conducted for each health outcome. As summarized in Table 4, all models exhibited statistically significant positive spatial autocorrelation (*p* < 0.001). The Moran’s I values ranged from 0.0677 (Cancer) to 0.1613 (mental health). While these values are close to zero and therefore indicate only weak spatial autocorrelation in magnitude, their associated z-scores were very large and statistically significant (*p* < 0.001). This combination shows that the residuals are not randomly distributed but instead exhibit a consistent, albeit modest, tendency for similar values to cluster geographically (see Supplementary Figures S1–S7). In other words, even weak spatial dependence becomes meaningful when detected across many census tracts. This non-random structure in OLS residuals points to underlying spatial processes not captured by global models and supports the application of spatially adaptive techniques such as Geographically Weighted Regression (GWR) and Multiscale GWR (MGWR) to better account for spatial heterogeneity in the relationships between health outcomes and explanatory variables.

**Table 4:** Residuals for spatial dependence.

Outcome	Moran’s I	z-score	*p*-value
Mental health	0.1613	23.01	<0.001
Diabetes	0.1393	19.87	<0.001
Blood pressure	0.1045	14.95	<0.001
Asthma	0.1021	14.61	<0.001
Stroke	0.094	13.49	<0.001
COPD	0.0714	10.28	<0.001
Cancer	0.0677	9.76	<0.001

### 3.3 Spatial analysis using GWR

Table 5 summarizes the statistical results from the GWR models (results for other years are included in the Supplementary material). At the same time, Figure 3 shows the spatial distribution of local R^2^ values across Harris County for the seven health outcomes. Overall, the GWR models improved upon the global OLS regressions, yielding higher adjusted R^2^ values (e.g., for blood pressure, R^2^ improved from 0.567 to 0.715; for cancer from 0.576 to 0.661; and for diabetes from 0.741 to 0.835). Similarly, AICc values decreased substantially across outcomes, highlighting the enhanced performance of GWR relative to OLS, although the models remained less parsimonious than MGWR, as discussed in the next section.

**Table 5:** Statistical modeling results using GWR (2022 data).

Outcome	Statistic	Housing age	Poverty rate	Unemployment rate	Uninsured rate	Impervious surface	Ozone	PM_2.5_
Blood pressure	Mean	0.2102	0.4875	0.1214	−0.299	−0.2265	−0.1127	0.1051
	Median	0.1988	0.4634	0.0891	−0.305	−0.1862	−0.2711	0.1302
	SD	0.1725	0.1947	0.1429	0.0021	0.1808	0.3246	0.4057
	Adjusted R^2^	0.715						
	AICc	2,341						
Cancer	Mean	0.4287	−0.1443	0.0285	−0.4892	−0.1768	0.5885	0.2879
	Median	0.4022	−0.1521	0.0281	−0.4914	−0.1427	0.6011	0.2484
	SD	0.1889	0.1418	0.0025	0.1162	0.2097	0.0451	0.5392
	Adjusted R^2^	0.661						
	AICc	1957						
Asthma	Mean	−0.005	0.692	0.1513	−0.0471	−0.0594	−0.1889	−0.0627
	Median	−0.009	0.673	0.1634	−0.0515	−0.0318	−0.0387	0.0193
	SD	0.0733	0.1769	0.0977	0.0611	0.1415	0.5664	0.5992
	Adjusted R^2^	0.793						
	AICc	1,240						
COPD	Mean	0.264	0.6812	0.0928	−0.1761	−0.1292	0.5016	0.5175
	Median	0.261	0.6618	0.0719	−0.1788	−0.1517	0.546	0.5231
	SD	0.0112	0.1874	0.0896	0.0063	0.1827	0.0892	0.2591
	Adjusted R^2^	0.781						
	AICc	1,453						
Diabetes	Mean	0.1593	0.4667	0.0721	−0.0752	−0.1408	0.0311	0.4298
	Median	0.1542	0.4623	0.0648	−0.0771	−0.1182	0.0342	0.5324
	SD	0.1325	0.2498	0.0624	0.0748	0.1242	0.1164	0.4517
	Adjusted R^2^	0.835						
	AICc	1,375						
Mental health	Mean	0.0532	0.6881	0.0389	0.2036	0.0182	0.1723	0.0991
	Median	0.0571	0.6814	0.0317	0.1995	0.0197	0.1558	0.1883
	SD	0.0521	0.0945	0.0381	0.1352	0.0246	0.0712	0.3341
	Adjusted R^2^	0.864						
	AICc	971						
Stroke	Mean	0.1864	0.5237	0.1265	−0.2014	−0.1372	0.2914	0.6159
	Median	0.1822	0.472	0.0811	−0.2009	−0.1087	0.2788	0.7081
	SD	0.0021	0.2565	0.1418	0.0287	0.1504	0.2247	0.3189
	Adjusted R^2^	0.771						
	AICc	1,208						

**Figure 3:**
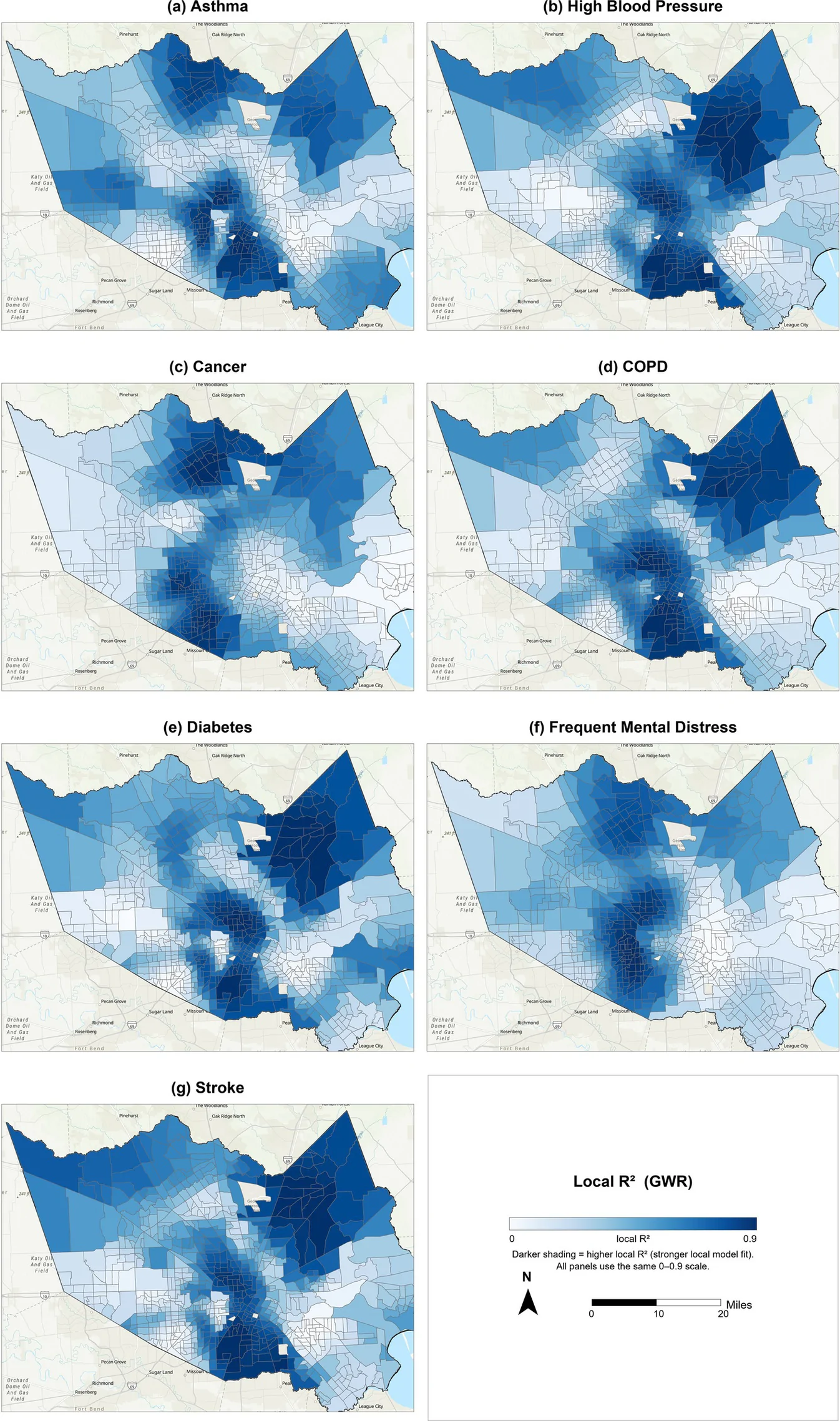
Spatial distribution of local R2 for GWR model results. Census tracts in pink represent areas with no data; the gradient color in the legend shows the local R^2^ in descending order (darker to lighter colors for highest to lowest). **(a)** asthma, **(b)** high blood pressure, **(c)** cancer, **(d)** COPD, **(e)** diabetes, **(f)** frequent mental distress, **(g)** stroke.

**Figure 4:**
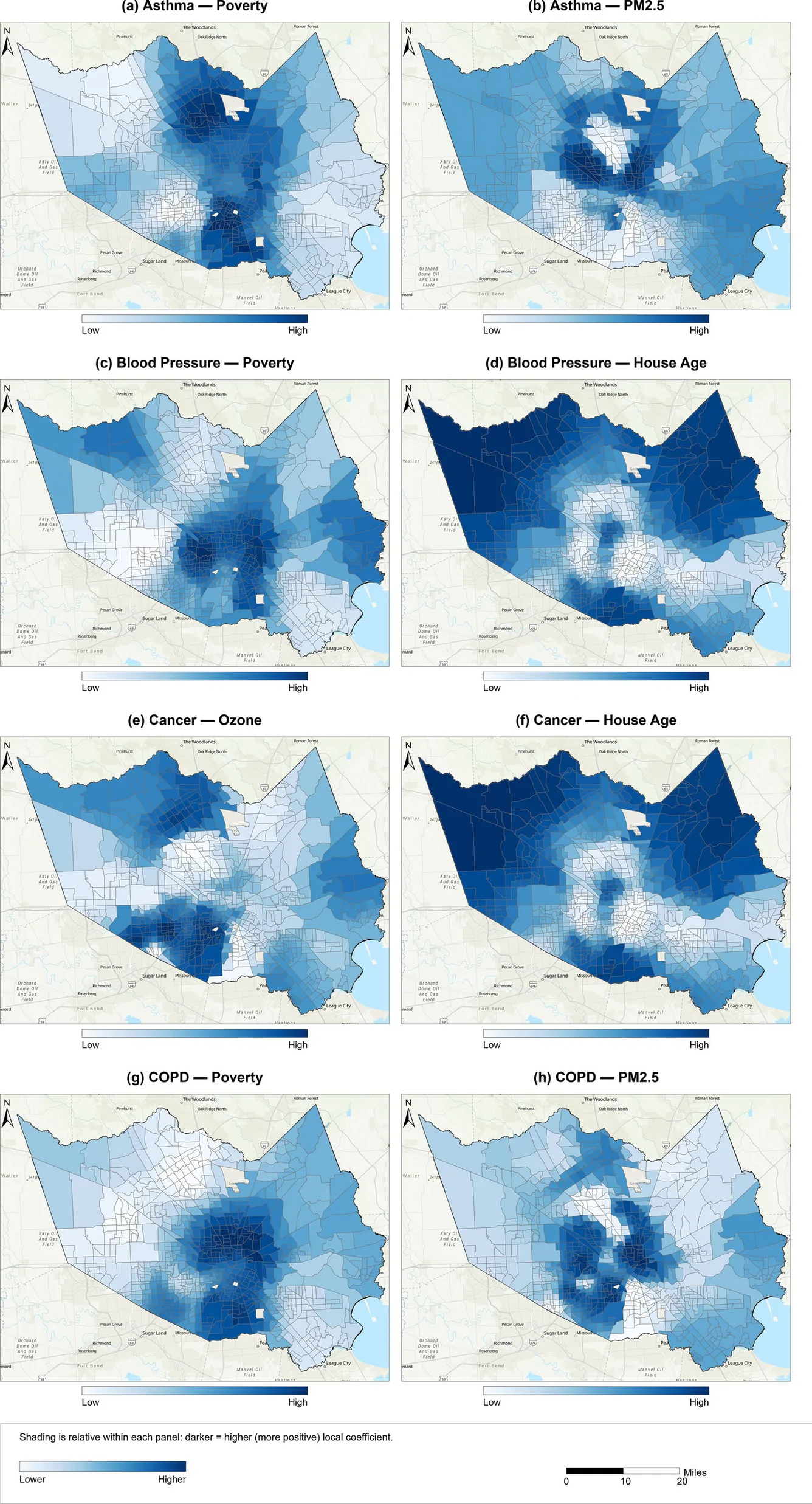
Spatial distribution of GWR coefficient estimates for selected key explanatory variables for asthma, blood pressure, cancer, and COPD health outcomes in Harris County. **(a)** asthma-poverty, **(b)** asthma-PM2.5, **(c)** blood pressure-poverty, **(d)** blood pressure-house age, **(e)** cancer-ozone, **(f)** cancer-house age, **(g)** COPD-poverty, **(h)** COPD-PM2.5.

Because GWR produces a distribution of local coefficients rather than a single global estimate, Table 5 also reports the mean, median, and standard deviation (SD) for each predictor. These summary statistics illustrate the extent of spatial heterogeneity in predictor effects. For example, in the blood pressure model, poverty exhibited a mean local coefficient of 0.49 (median = 0.46, SD = 0.19), confirming a consistently positive association but with notable spatial variation in its strength across the county. Similarly, in the cancer model, ozone showed a mean coefficient of 0.59 with a small SD (0.05), indicating a stable positive effect, whereas PM_2.5_ displayed a larger SD (0.54), reflecting greater geographic variability. Such differences highlight predictors that exert spatially uniform effects versus those with heterogeneous, location-specific influences.

Spatially, Figures 3–5 illustrate the geospatial modeling results for GWR. Figure 3 shows that local R^2^ values were highest in the central and southeastern regions of Harris County, which cover the urbanized core of Houston. These areas consistently exhibited stronger model fits across outcomes, suggesting more stable and spatially structured relationships between health indicators and explanatory variables. In contrast, the northern and western peripheries displayed lower local R^2^ values, indicating weaker spatial dependence and the influence of unobserved or more heterogeneous contextual factors. Thus, GWR improved upon OLS by capturing spatial heterogeneity; however, its explanatory power and efficiency were more modest than those of MGWR, as discussed in the following sections.

**Figure 5:**
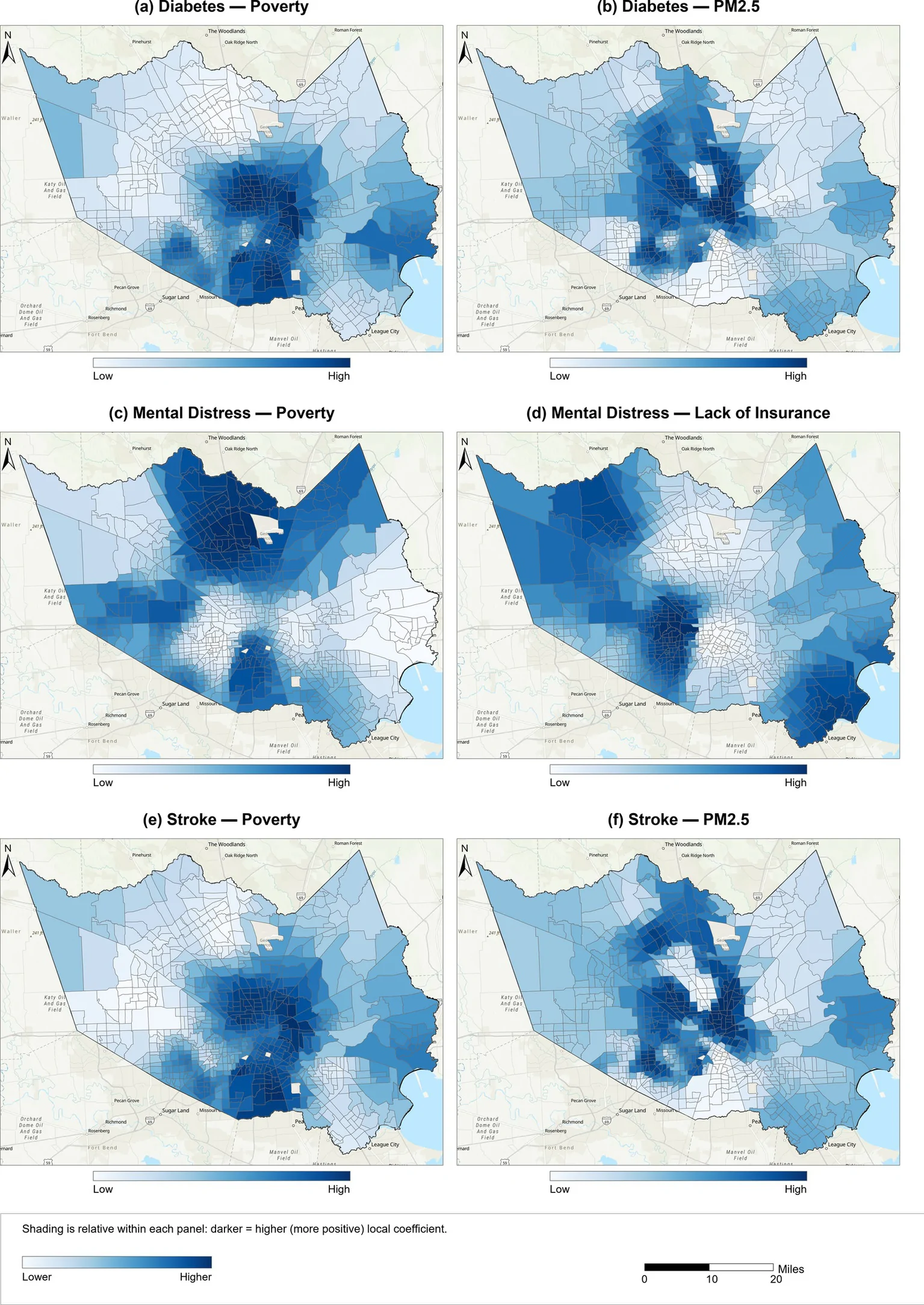
Spatial distribution of GWR coefficient estimates for selected key explanatory variables for diabetes, frequent mental distress, and stroke health outcomes in Harris County. **(a)** diabetes-poverty, **(b)** diabetes-PM2.5, **(c)** mental distress-poverty, **(d)** mental distress-lack of insurance, **(e)** stroke-poverty, **(f)** stroke-PM2.5.

### 3.4 Spatial analysis using MGWR

Table 6 summarizes the statistical output of MGWR (tables for other years are presented in the Supplementary material), and Figure 6 presents the spatial distribution of local R^2^ values for all seven health indicators in Harris County. Overall, the MGWR model provided substantial improvements over the global OLS model, as evidenced by consistently higher adjusted R^2^ values and lower AIC_c_ scores. For instance, the adjusted R^2^ improved from 0.567 to 0.753 for blood pressure and from 0.576 to 0.695 for cancer. AICc values also decreased considerably across all models, reinforcing the improved model performance. For example, the AICc dropped from 1,524 to 812 for asthma and from 1,697 to 1,008 for stroke, suggesting greater model parsimony and better fit. Similar improvements were observed for all other health outcomes, underscoring the superior explanatory power of MGWR in accounting for spatial non-stationarity. As MGWR produces local coefficients for each predictor, Table 6 also reports the mean, median, and standard deviation of these distributions. The reported statistics highlight both the overall direction of predictor effects and the degree of spatial heterogeneity. For example, in the diabetes model, poverty showed a strong positive mean coefficient (0.51) with a relatively large SD (0.22), indicating a consistent but spatially variable effect across Harris County. In contrast, ozone in the cancer model exhibited a mean coefficient of 0.62 with a small SD (0.03), suggesting that its association with cancer prevalence was both positive and spatially stable. Such contrasts illustrate which predictors exert broad, uniform effects and which operate in more localized or context-specific ways.

**Table 6:** Statistical analysis results of MGWR estimation.

Outcome	Statistic	Housing age	Poverty rate	Unemployment rate	Uninsured rate	Impervious surface	Ozone	PM_2.5_
Blood pressure	Mean	0.2333	0.5227	0.1392	−0.33	−0.2432	−0.1381	0.1193
	Median	0.2277	0.4993	0.1003	−0.33	−0.2037	−0.2968	0.1445
	SD	0.1476	0.17	0.1255	0.0006	0.1559	0.2859	0.3721
	Adjusted R^2^	0.753						
	AICc	1,221						
Cancer	Mean	0.4561	−0.1587	0.0329	−0.5185	−0.1987	0.6214	0.3133
	Median	0.4168	−0.1642	0.0327	−0.5213	−0.1639	0.6223	0.2737
	SD	0.1665	0.1256	0.0011	0.1005	0.1815	0.0319	0.4923
	Adjusted R^2^	0.695						
	AICc	1,373						
Asthma	Mean	0.003	0.725	0.1645	−0.054	−0.0728	−0.2077	−0.0856
	Median	−0.001	0.7006	0.1779	−0.0601	−0.0435	−0.0511	0.0362
	SD	0.0612	0.1514	0.0848	0.0535	0.1234	0.5248	0.5538
	Adjusted R^2^	0.854						
	AICc	812						
COPD	Mean	0.293	0.7171	0.1045	−0.1917	−0.142	0.5308	0.5593
	Median	0.292	0.6993	0.0842	−0.192	−0.1618	0.571	0.5589
	SD	0.0093	0.1613	0.0779	0.0046	0.1646	0.0748	0.2324
	Adjusted R^2^	0.822						
	AICc	952						
Diabetes	Mean	0.1798	0.5065	0.0812	−0.0831	−0.1563	0.0396	0.4747
	Median	0.1799	0.5051	0.0721	−0.0842	−0.133	0.0405	0.5807
	SD	0.1179	0.219	0.0552	0.0664	0.1094	0.0989	0.4161
	Adjusted R^2^	0.879						
	AICc	694						
Mental health	Mean	0.0619	0.7228	0.0453	0.2257	0.0215	0.1857	0.1177
	Median	0.0659	0.7159	0.0361	0.2217	0.023	0.1702	0.2074
	SD	0.045	0.0819	0.0323	0.1196	0.0207	0.0625	0.3034
	Adjusted R^2^	0.910						
	AICc	427						
Stroke	Mean	0.2083	0.5638	0.1394	−0.2175	−0.1541	0.3211	0.6716
	Median	0.2083	0.507	0.0915	−0.2165	−0.1211	0.3066	0.7737
	SD	0.0006	0.2257	0.125	0.0239	0.1326	0.1992	0.2926
	Adjusted R^2^	0.810						
	AICc	1,008						

**Figure 6:**
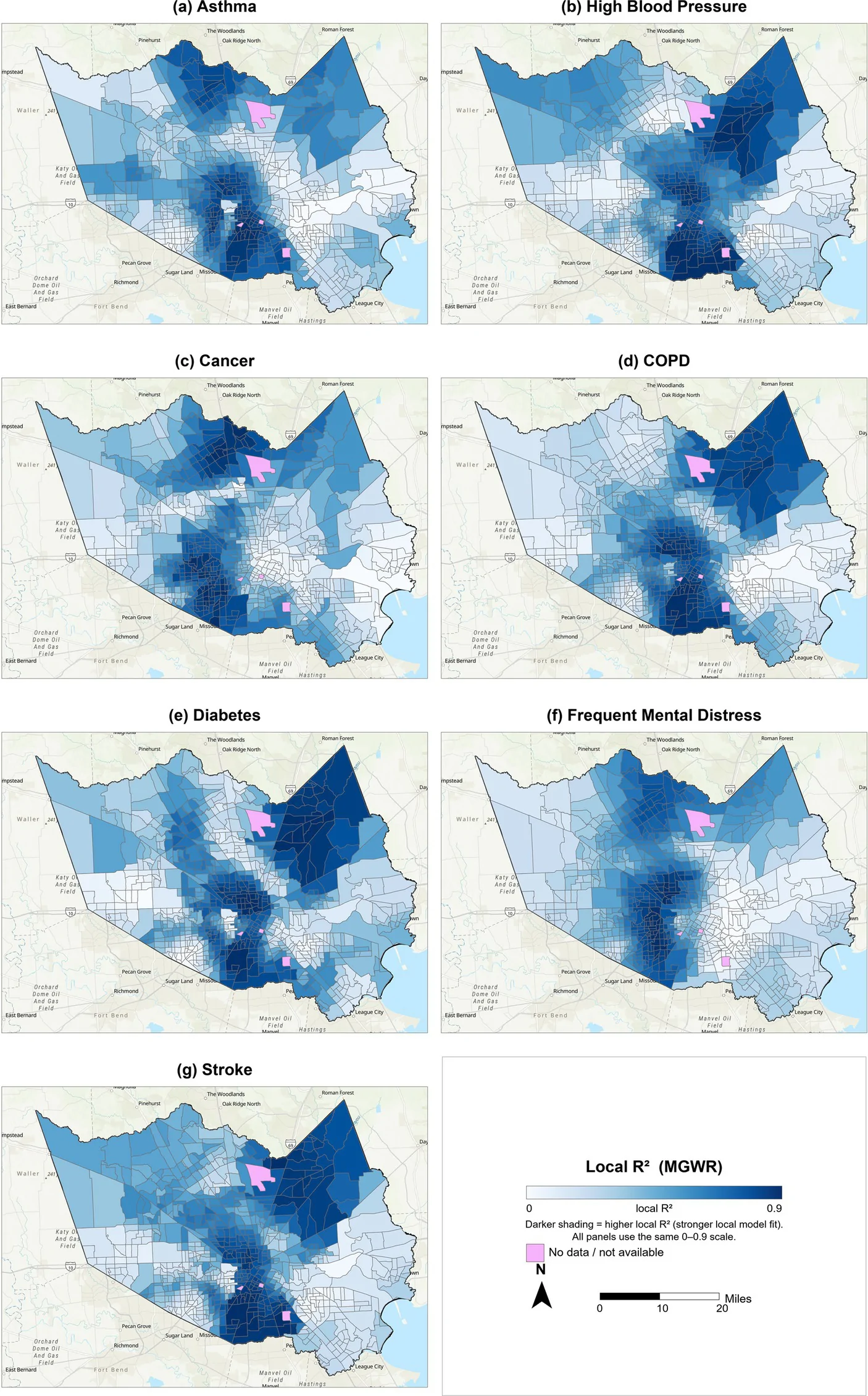
Spatial distribution of local *R*2 for the MGWR model results. Note: Census tracts in pink represent areas with no data; the gradient color in the legend shows the local *R*2 in descending order (darker to lighter colors for highest to lowest). **(a)** asthma, **(b)** high blood pressure, **(c)** cancer, **(d)** COPD, **(e)** diabetes, **(f)** frequent mental distress, **(g)** stroke.

Spatially, Figure 6 highlights that the findings that high local R^2^ values were generally concentrated in the central and southeastern portions of Harris County, which largely encompass urban areas within and around Houston. These regions demonstrated stronger model fits, indicating that the relationships between predictors and health outcomes were more consistent and spatially structured in the urban core. In contrast, peripheral regions, particularly in the northern and western parts of the county, exhibited lower local R^2^ values, suggesting weaker spatial dependence or more heterogeneous, possibly unevidenced, drivers of health outcomes in the less urbanized fringe of the county.

When comparing outcomes, mental health had the highest adjusted R^2^ (0.910), followed by diabetes (0.879) and asthma (0.854), suggesting that the chosen predictors were most effective at explaining spatial variation for these diseases. Meanwhile, cancer (0.695) and stroke (0.810) showed relatively lower adjusted R^2^ values, pointing to the potential influence of unrepresented factors in these outcomes.

The spatial patterns of the coefficients of key explanatory variables for each health outcome are discussed in the next section. To aid interpretation, only the areas with statistically significant local estimates (*p* < 0.1) are visualized and discussed using the coefficient maps.

To effectively interpret the spatial variability of explanatory factors across health outcomes, a subset of key variables was selected for visualizing local coefficient estimates. Rather than presenting maps for all predictors, which would be both visually overwhelming and analytically redundant, the presentation focuses on variables that exhibit high spatial variation, statistical significance across a large percentage of features, and clear relevance to public health or environmental policy. For each health outcome, two variables were prioritized that best demonstrated spatial heterogeneity and interpretive value.

The spatial coefficient analysis results for the seven health outcomes are visualized in Figures 7, 8. These maps illustrate the spatial heterogeneity of the associations between key explanatory variables and health outcomes across Harris County, Texas. As reflected in these results, poverty consistently emerged as a critical determinant, with positive associations for all health indicators. For example, the poverty coefficient for asthma and diabetes was especially high in central and southeastern parts of the county, indicating that areas with higher poverty rates were also associated with a greater prevalence of these conditions. A similar pattern was observed for blood pressure, stroke, and mental health outcomes, underscoring the persistent role of socioeconomic status in shaping spatial health variation within Harris County.

**Figure 7:**
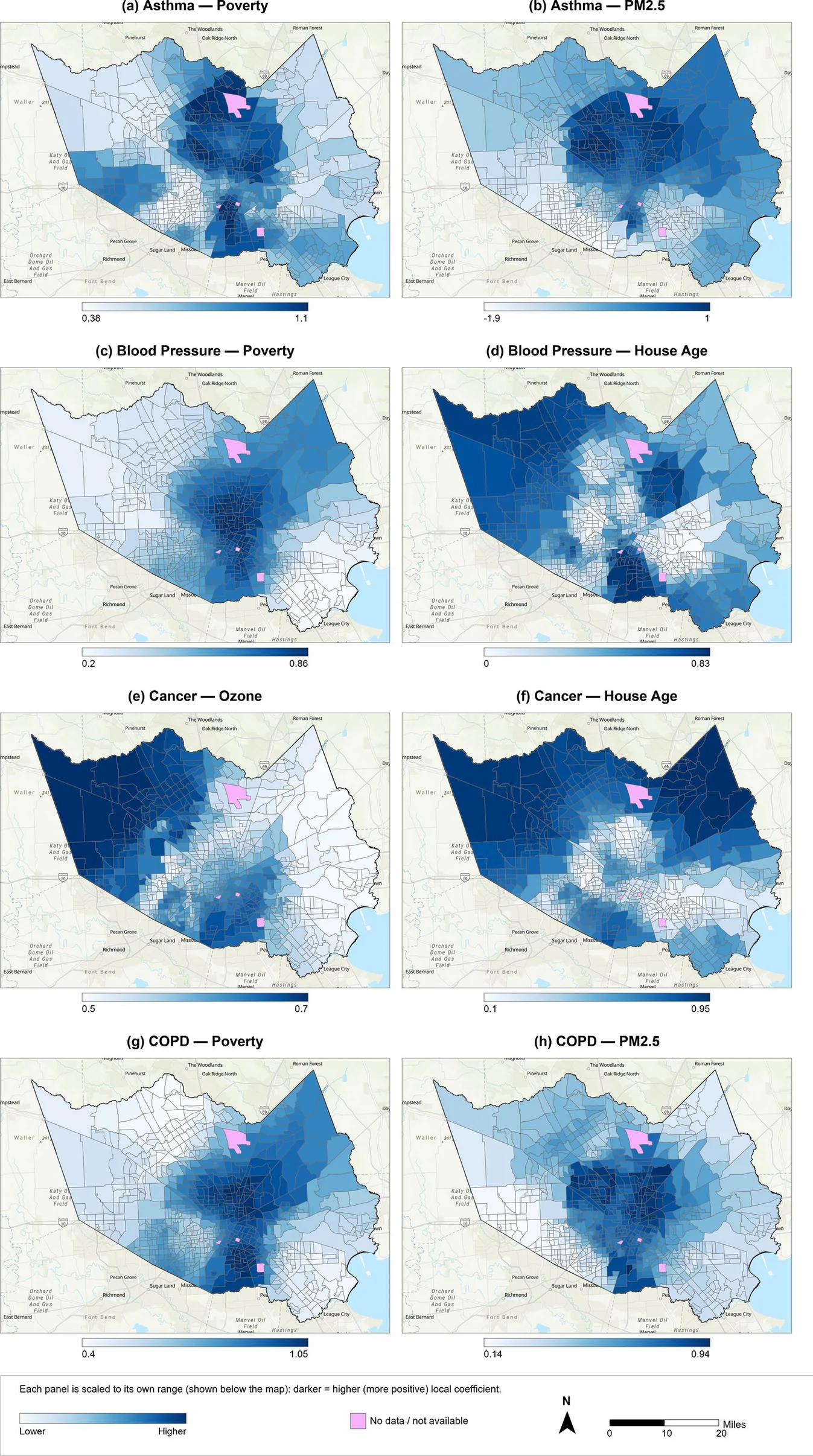
Spatial distribution of MGWR coefficient estimates for selected key explanatory variables for asthma, blood pressure, cancer, and COPD health outcomes in Harris County. **(a)** asthma-poverty, **(b)** asthma-PM2.5, **(c)** blood pressure-poverty, **(d)** blood pressure-house age, **(e)** cancer-ozone, **(f)** cancer-house age, **(g)** COPD-poverty, **(h)** COPD-PM2.5.

**Figure 8:**
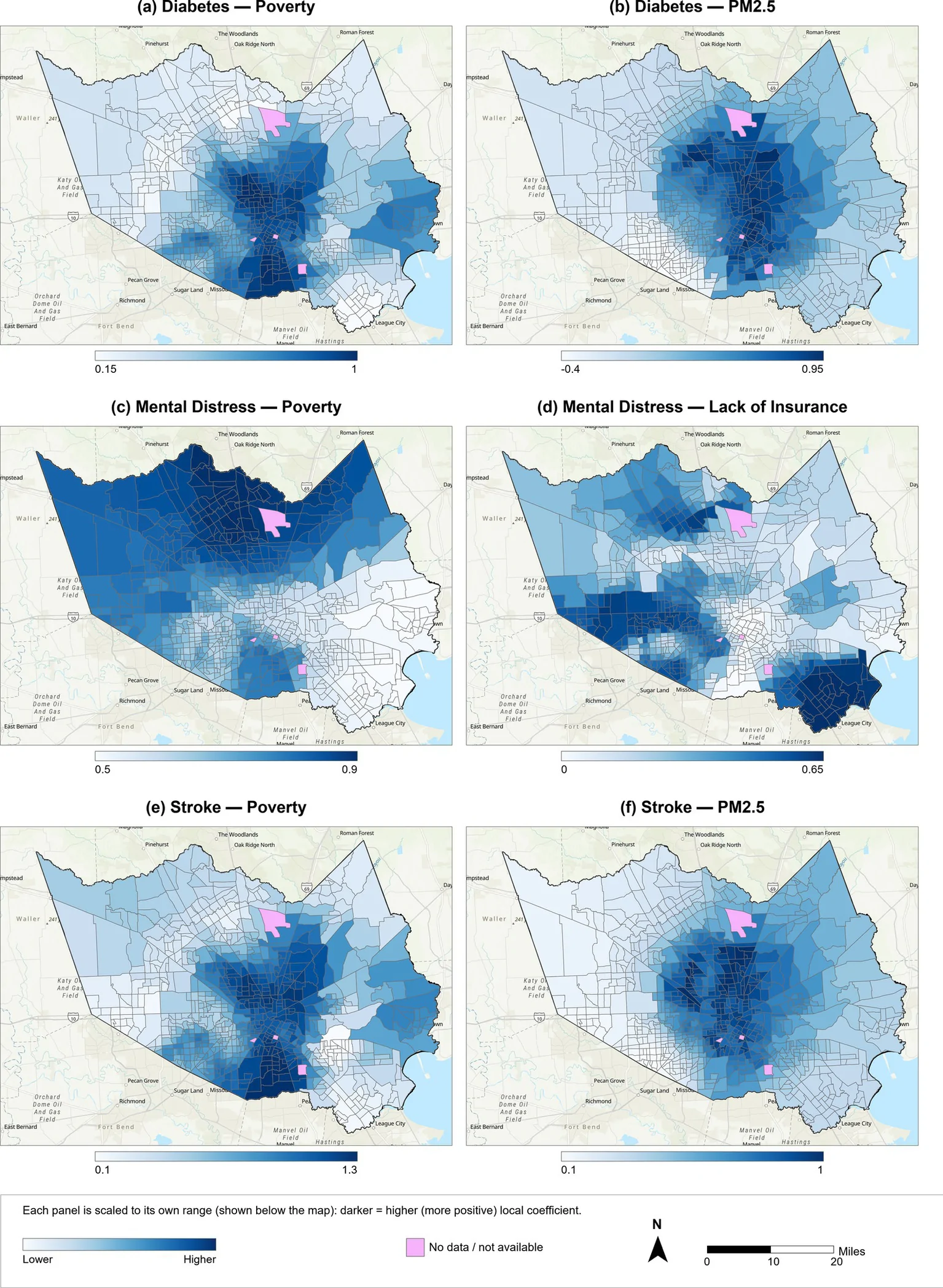
Spatial distribution of MGWR coefficient estimates for selected key explanatory variables for diabetes, frequent mental distress, and stroke health outcomes in Harris County. **(a)** diabetes-poverty, **(b)** diabetes-PM2.5, **(c)** mental distress-poverty, **(d)** mental distress-lack of insurance, **(e)** stroke-poverty, **(f)** stroke-PM2.5.

PM_2.5_ exposure was another dominant predictor, exhibiting strong positive associations with asthma, COPD, diabetes, and stroke. In all four cases, spatial clusters of high PM_2.5_ coefficients were observed in eastern and central census tracts—areas with historically higher air pollution levels due to proximity to industrial corridors. This suggests a robust geospatial link between air quality and chronic health conditions, particularly respiratory and cardiovascular outcomes.

Housing age was particularly influential in explaining cancer and blood pressure. Both outcomes showed stronger associations in older neighborhoods—mainly in the northeastern and southeastern quadrants of the county—possibly reflecting legacy environmental exposures or deteriorating housing conditions.

For mental health outcomes, the rate of uninsured individuals showed positive spatially varying relationships, particularly in southern and eastern Harris County, implying barriers to care access as a significant factor.

Ozone levels were found to be significantly associated with cancer, showing positive coefficients in a wide swath of the eastern half of the county. This may reflect long-term cumulative exposure patterns that vary across urban and suburban zones.

The visualizations in Figures 7, 8 confirm the spatial non-stationarity of social and environmental determinants of health, reinforcing the advantages of MGWR over global models. The direction and strength of associations varied spatially, and several variables demonstrated highly localized effects that were not captured by OLS or conventional GWR models.

### 3.5 OLS compared to GWR

To assess the benefits of incorporating spatial heterogeneity, model performance was first compared between OLS and GWR (Figure 9). Across all health outcomes, GWR yielded higher adjusted R^2^ values than OLS, indicating improved explanatory power when spatial non-stationarity was accounted for. For example, the adjusted R^2^ for blood pressure increased from 0.567 (OLS) to 0.715 with GWR, while the adjusted R^2^ for cancer improved from 0.576 to 0.661. Similar gains were observed for asthma (0.748–0.793), diabetes (0.741–0.835), and stroke (0.658 to 0.771). Mental health achieved the highest explanatory power under GWR, with an adjusted R^2^ of 0.864.

**Figure 9:**
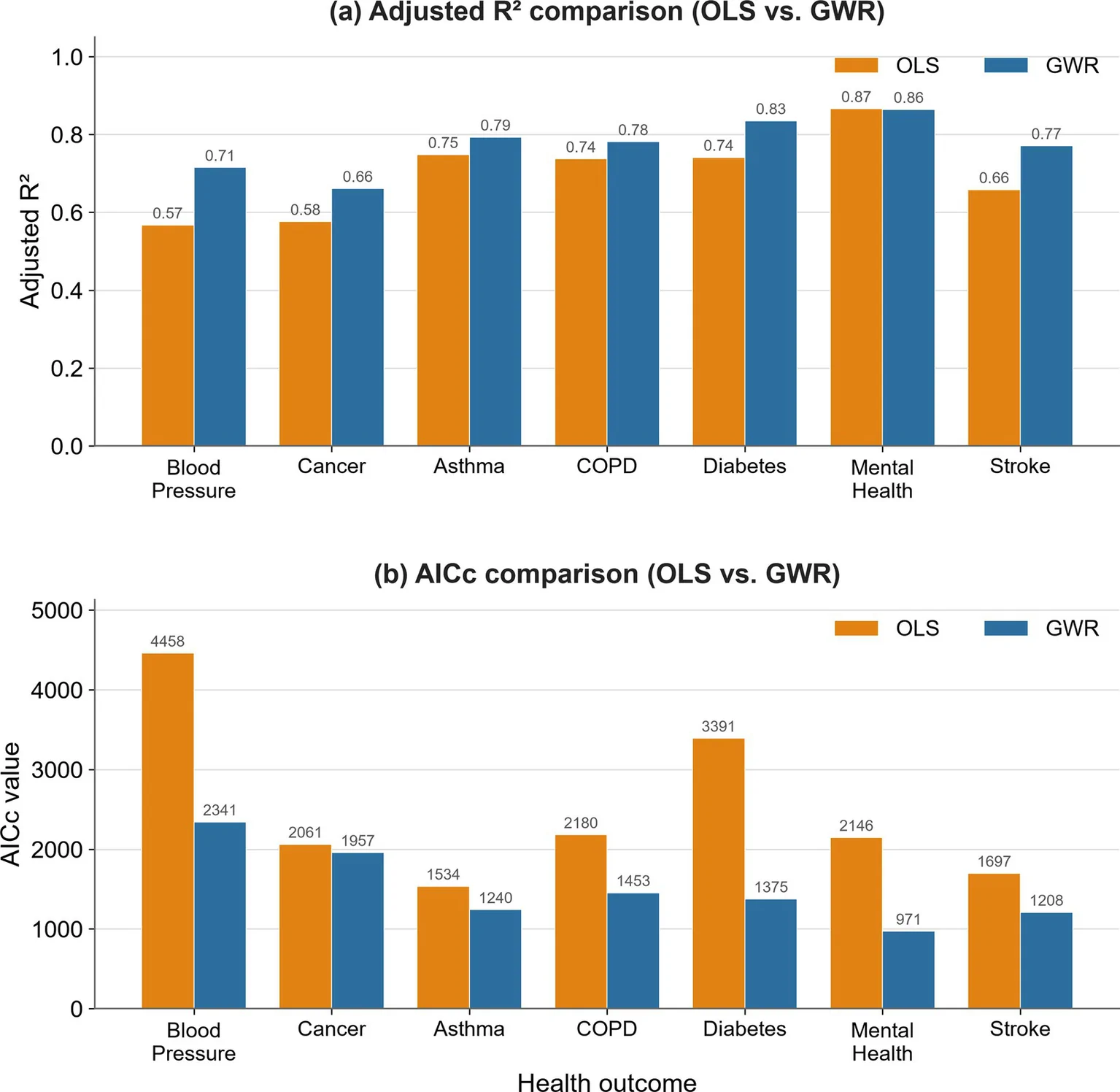
Comparison of OLS and GWR model performance across health outcomes. **(a)** adjusted R^2^ and **(b)** AICc for each health outcome.

In addition, AICc values consistently decreased under GWR, indicating more efficient model fits. For instance, the AICc dropped from 4458 (OLS) to 2341 (GWR) for blood pressure, from 2061.9 to 1956.6 for cancer, from 1,535 to 1,240 for asthma, and from 1,697 to 1,208 for stroke. These reductions confirm that GWR more effectively accounts for spatial dependence than OLS, although its reliance on a single bandwidth for all predictors may limit flexibility.

### 3.6 GWR compared to MGWR

Building on the improvements of GWR, MGWR further enhanced model performance by allowing each predictor to vary at its own spatial scale (Figure 10). Adjusted R^2^ values were uniformly higher for MGWR than GWR, underscoring the added benefit of multiscale modeling. For example, blood pressure improved from 0.715 (GWR) to 0.753 (MGWR), and cancer from 0.661 to 0.695. Diabetes and asthma also showed notable gains, reaching 0.879 and 0.854, respectively.

**Figure 10:**
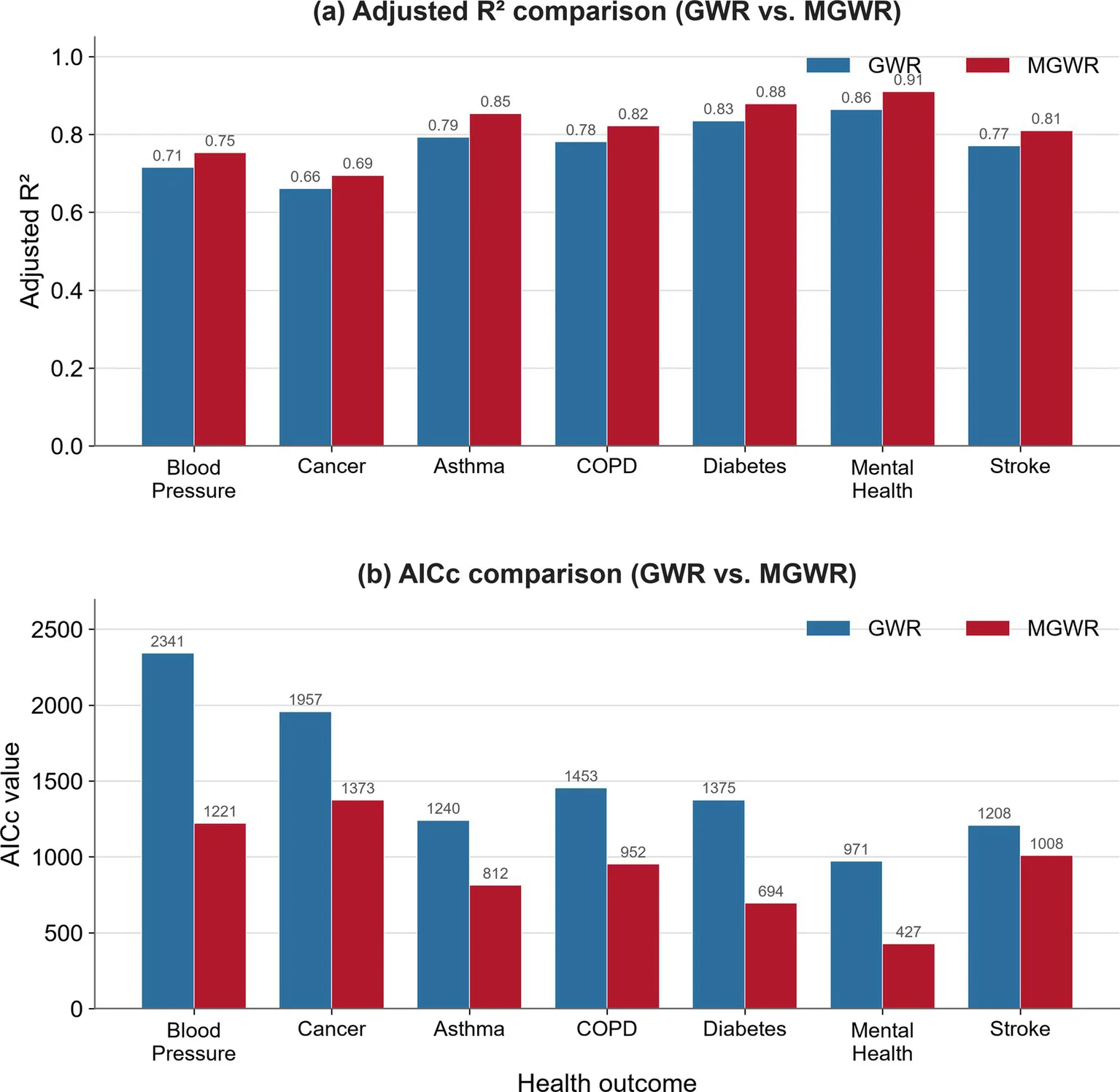
Comparison of GWR and MGWR model performance across health outcomes. **(a)** adjusted R^2^ and **(b)** AICc for each health outcome.

Similarly, AICc values were lower for MGWR across all outcomes, confirming its superior model fit. The improvement was particularly evident in stroke and mental health, where the multiscale approach captured spatial variation more effectively than a single bandwidth model. Compared with GWR, the local R^2^ surfaces under MGWR were more distinct and less diffuse, highlighting the advantage of modeling predictors at their appropriate scales.

Overall, while GWR represented a substantial improvement over OLS by capturing spatial heterogeneity, MGWR provided the most accurate and efficient framework by capturing variable-specific spatial processes.

### 3.7 Robustness of modeling for 2020 through 2024

To further assess the temporal robustness of the observed spatial relationships, the MGWR and OLS models were run for 2020, 2021, 2023, and 2024 using the same methodological framework and variables as in the 2022 analysis. Overall, the results demonstrated consistent patterns in model fit, predictor significance, and coefficient directionality. While minor year-to-year variations were expected due to fluctuations in the underlying data, the spatial structure of key associations—such as those linking poverty, air pollution, and uninsured rates to adverse health outcomes—remained stable. This temporal consistency demonstrates the reliability of the findings and reinforced their relevance for long-term spatial health planning. Detailed outputs for each year, including full OLS, GWR, and MGWR coefficient summaries, are provided in the Supplementary Tables S1–S12.

The OLS models confirm that the statistical associations identified in the main 2022 analysis are stable and reproducible. The direction and significance of all major predictors remain consistent across years, suggesting that the global relationships are not driven by short-term fluctuations or random variations in the data. In every year, socioeconomic indicators such as poverty and unemployment retain positive and significant coefficients, while the uninsured rate and impervious surface preserve their expected negative effects. These recurring patterns suggest that the global OLS relationships reflect systematic social and environmental gradients rather than year-specific noise.

From a performance standpoint, the temporal stability of Adjusted R^2^ and AICc values further supports the reliability of the OLS results (Supplementary Tables S1–S4). Using a goodness-of-fit consistency check,

$$\Delta R^{2}=R_{\mathrm{SI}}^{2}-R_{\text{Main}}^{2}\;\;\;\;\text{ΔAICc}=\mathrm{AIC}c_{\mathrm{SI}}-\mathrm{AIC}c_{\text{Main}}$$

Year-to-year deviations were compared in model fit relative to 2022. For all outcomes, ∣ΔR^2^∣ remained ≤ 0.09 and ∣ΔAICc∣ ≤ 110, confirming that differences across years are statistically minor and within the range expected for models of this scale. The small variation in R^2^ across years (typically within ±0.05 for each outcome) indicates that the model’s explanatory power is nearly identical from 2020 to 2024. The consistent ranking of model fit among health outcomes also confirms that relative explanatory contributions are structurally unchanged. Spatial diagnostics show weak but statistically significant residual clustering in the same geographic areas across time, implying that spatial dependence is persistent but limited. Altogether, the OLS results provide an internally consistent foundation on which the spatial models are built. Their temporal reproducibility validates the global structure of relationships that GWR and MGWR later refine at local scales.

The GWR results (Supplementary Tables S5–S8) across the 5 years confirm both the stability and credibility of the spatial patterns reported for 2022. In each year, GWR improves model performance over OLS by a similar magnitude, indicating that the spatially varying relationships are not unique to a single dataset but are repeatable over time. The observed increases in Adjusted R^2^ (approximately +0.10–0.15) and reductions in AICc are remarkably consistent from 2020 through 2024, validating that introducing spatial heterogeneity systematically strengthens explanatory performance. Consistent with the validation framework described above, year-to-year deviations in ΔR^2^ and ΔAICc remained well below the stability thresholds, indicating that GWR performance metrics are reproducible across time.

The coefficient distributions in the Supplementary material tables demonstrate reproducibility in both direction and variability. For example, poverty, unemployment, and the uninsured rate maintain positive associations with nearly identical mean and median coefficients across years, confirming that the social determinants of health act similarly over time. The standard deviations of local coefficients also remain within narrow ranges, indicating that the degree of spatial variation itself is stable. Environmental predictors such as ozone and PM₂.₅ continue to exhibit location-dependent behavior with comparable variability across years, thereby validating that these spatial patterns are genuine rather than random.

Spatially, the local R^2^ distributions exhibit the same core–periphery configuration every year: Stronger model fits in central and southeastern Harris County, and weaker fits in peripheral zones. This geographic consistency provides strong validation that the spatial heterogeneity captured by GWR represents persistent structural conditions rather than artifacts of any specific year. Therefore, the multi-year GWR analyses confirm that the 2022 spatial patterns are temporally reliable and that spatial non-stationarity is an enduring feature of the system

The MGWR analyses (Supplementary Tables S9–S12) further validate the robustness of the main models by confirming that the multi-scale spatial processes observed in 2022 persist across all other years. The improvements in Adjusted R^2^ and AICc relative to GWR are observed across all datasets, with similar magnitudes and rankings among outcomes. When evaluated using the same goodness-of-fit consistency framework ΔR^2^ and ΔAICc, year-to-year deviations again fall below the thresholds, supporting the temporal reproducibility of the MGWR performance improvements. This temporal repeatability demonstrates that the MGWR framework captures stable and meaningful multi-scale relationships rather than overfitting to a single year.

The consistency of coefficient statistics across time provides additional evidence of validation. For instance, the poverty rate remains the most influential predictor across all years, with mean values around 0.45–0.75 and small temporal fluctuations. PM_2.5_ coefficients also remain positive for respiratory and cardiometabolic outcomes with nearly constant means and standard deviations. Ozone’s association with cancer stays positive and spatially uniform across all years, confirming that the observed exposure–response relationship is persistent. These patterns collectively show that the variable-specific bandwidths estimated by MGWR are stable and reproduce comparable spatial scales of influence through time.

Spatially, MGWR local R^2^ patterns remain nearly identical across 2020–2024. High model fits persist in the central and southeastern portions of Harris County, coinciding with the urban and industrialized regions where both social stressors and environmental exposures are highest. This long-term spatial coherence confirms that the MGWR results are not limited to a single temporal snapshot but rather reflect structural, long-lived disparities. In addition, the repeated AICc reductions—often 300–500 points lower than OLS—demonstrate that MGWR achieves this robustness with greater model parsimony, reinforcing its validity as the most efficient representation of spatial processes among the three approaches.

## 4 Discussion

The relationships between environmental, built-environment, and socioeconomic variables and chronic health outcomes in Harris County demonstrated significant spatial heterogeneity, as captured by the MGWR model. These spatial variations underscore the limitations of conventional global regression approaches, such as OLS, and highlight the importance of applying multiscale spatial methods to uncover nuanced patterns of health determinants across urban environments.

Consistent with the proposed hypotheses in this study, the directions of the majority of the predictors aligned with existing literature. Poverty, for instance, was universally associated with higher prevalence of all seven chronic health outcomes. The positive coefficients of poverty were particularly pronounced in central and southeastern portions of Harris County, reinforcing the spatial concentration of health burdens in socioeconomically challenged areas. Unemployment and lack of insurance—two other indicators of socioeconomic status—also showed positive associations for the majority of the conditions, although their spatial impacts varied more locally.

Among the environmental predictors, PM_2.5_ exposure was one of the most influential variables. PM_2.5_ showed consistently strong positive associations with asthma, COPD, diabetes, and stroke—conditions that are well-documented to be exacerbated by fine particulate air pollution. Spatially, tracts in the eastern part of Harris County—near major highways and industrial corridors—exhibited the highest PM_2.5_ coefficients, reflecting historical exposure patterns. These findings corroborate studies linking elevated ambient particulate matter levels to increased respiratory and cardiovascular risk (1, 2).

Ground-level ozone also demonstrated significant associations, albeit more selectively. Ozone levels were a strong predictor of cancer prevalence, with higher coefficients appearing in densely populated eastern neighborhoods. The observed spatially variable pattern aligns with cumulative ozone exposure from mobile and industrial sources concentrated near the Houston Ship Channel. Interestingly, ozone was also positively associated with mental health outcomes, echoing prior research that suggests potential neurological and psychological impacts of prolonged ozone exposure.

Built-environment characteristics, such as housing age and imperviousness, provided further insights. Older housing stock showed strong positive associations with cancer and blood pressure outcomes, likely due to long-term environmental exposures or infrastructure degradation. Impervious surface area, used as a proxy for urban intensity, showed mixed associations with the health outcomes studied. In the case of diabetes and stroke, the results showed negative coefficients in certain tracts—possibly reflecting complex interactions among land use, green space, and socioeconomic context. However, its relatively lower significance and consistency across models suggest that its influence may be mediated or masked by other factors.

One notable finding was the variable role of lack of health insurance. For mental health outcomes, uninsured rates emerged as a particularly strong predictor, suggesting that access to care plays a critical role in mental health burden. This aligns with literature documenting variation in behavioral health access among uninsured populations (24). Additionally, the spatial variation in coefficients for the uninsured rate was higher than that of other socioeconomic variables, highlighting localized barriers to care across the county.

While the MGWR model improved explanatory power for all outcomes compared to OLS—as evidenced by increases in adjusted R^2^ and reductions in AICc values—the analysis also revealed areas of weaker model fit. Specifically, peripheral and less urbanized tracts in northern and western Harris County often exhibited lower local R^2^ values. This suggests that additional unevidenced factors—such as dietary patterns, cultural practices, or unobserved environmental exposures—may be at play in these areas.

It is also important to note that certain predictors, despite their expected influence, demonstrated weak or inconsistent relationships. For example, housing age and imperviousness were not statistically significant for asthma or COPD, which may indicate that short-term environmental conditions outweigh long-term built-environment factors for these two diseases. Similarly, ozone did not show significant associations with blood pressure or diabetes, which contrasts with some national-level studies and suggests the need for more granular exposure data or temporal matching in future studies.

Overall, the MGWR framework enabled a deeper understanding of spatially varying associations between health outcomes and their determinants. The ability to model each predictor at its optimal spatial scale is critical in heterogeneous urban regions such as Harris County, where localized socioeconomic and environmental gradients are pronounced. The spatial clustering of high-risk coefficients suggests potential targets for place-based interventions, particularly in eastern and southeastern neighborhoods with elevated poverty, pollution, and chronic disease prevalence.

The results not only reinforced existing public health concerns in socioeconomically challenged and environmentally burdened communities of Harris County but also highlighted the diverse spatial scales at which different determinants operate. By enabling variable-specific bandwidths, MGWR provided a more nuanced understanding of the multi-scalar nature of urban health risks. These insights can support spatially targeted interventions, resource allocation, and policy measures to mitigate chronic health burdens in urban areas.

Despite the study’s contributions, several limitations should be acknowledged. First, the analysis focused on area-level predictors at the census-tract scale, which may obscure individual-level variations in health outcomes and exposures. The modifiable areal unit problem (MAUP) may also influence the results, as different spatial units might yield different statistical associations. Additionally, while MGWR effectively captures spatial heterogeneity, it does not account for temporal dynamics. Although consistency of spatial associations across multiple years (2020–2024) was verified, the model was not designed to detect or explain temporal trends.

Another limitation is the exclusion of certain risk factors that influence chronic health outcomes, such as dietary habits, genetic predispositions, physical activity, and clinical biomarkers (e.g., cholesterol and blood glucose levels). These factors were unavailable at the spatial resolution of interest and thus could not be incorporated. The inclusion of such variables in future studies could enhance explanatory power and predictive accuracy. Moreover, although MGWR improves model fit and interpretability, it remains a descriptive tool and does not establish causality.

Future research might benefit from integrating temporal extensions of MGWR or combining spatial models with longitudinal health datasets to better understand temporal patterns. Additionally, incorporating machine learning techniques alongside spatial regression could offer new perspectives on the complex interactions among urban health determinants. Finally, the development of predictive spatial tools based on MGWR outputs could support proactive public health planning, especially in high-risk urban regions facing compounding environmental and social stressors.

## 5 Conclusion

This study combined OLS, GWR, and MGWR to map the relationships between environmental, built-environment, and socioeconomic factors and seven chronic health outcomes across the census tracts of Harris County, Texas. By allowing each predictor to operate at its own spatial scale, MGWR consistently outperformed the global OLS and single-bandwidth GWR models, producing the highest adjusted R^2^ and the lowest AICc for every outcome. Poverty and PM_2.5_ emerged as the most influential and spatially persistent predictors, with the heaviest burdens concentrated in the central, eastern, and southeastern tracts near industrial corridors and lower-income communities.

Re-estimating the framework for 2020, 2021, 2023, and 2024 reproduced the 2022 spatial patterns within narrow stability thresholds, indicating that the identified associations reflect persistent structural conditions rather than single-year artifacts. The framework is therefore useful not only for explaining current disparities but also for estimating the chronic disease burden in tracts or years where health data are missing or incomplete.

These findings should be interpreted as associational rather than causal and are subject to the area-level and modifiable areal unit limitations noted above. Future studies should incorporate individual-level and temporal data, additional risk factors such as diet, physical activity, and clinical biomarkers, and machine-learning approaches, and should translate the MGWR coefficient surfaces into decision-support tools for spatially targeted interventions. Overall, the study demonstrates that multiscale spatial modeling offers an actionable and transferable basis for identifying and prioritizing vulnerable communities in urbanized, industrialized regions such as Harris County.

## Data Availability

Publicly available datasets were analyzed in this study. Health-outcome estimates are from the CDC PLACES project (https://www.cdc.gov/places/). Ambient PM2.5 and ozone data are from the U.S. EPA Remote Sensing Information Gateway (RSIG), specifically the fused air-quality surfaces derived from the hierarchical Bayesian model (https://www.epa.gov/hesc/rsig-related-downloadable-data-files). Socioeconomic and housing-age variables are from the U.S. Census Bureau American Community Survey 5-Year Estimates (https://www.census.gov/programs-surveys/acs/). Percent impervious surface is from the Multi-Resolution Land Characteristics Consortium (https://www.mrlc.gov/). All datasets are publicly accessible at these sources.

## Supplementary material

Supplementary Tables S1-S12. Report the OLS, GWR, and MGWR coefficient estimates, significance, VIF, adjusted R^2^, and AICc for the robustness years (2020, 2021, 2023, 2024).

Supplementary Figures S1-S7. Present the OLS standardized-residual maps for high blood pressure (S1), cancer (S2), COPD (S3), asthma (S4), frequent mental distress (S5), diabetes (S6), and stroke (S7).
