# Supplementary material for: Geospatial modeling of environmental and socioeconomic drivers of health outcomes
Source: Front Public Health. 2026 Sep 14;14:1790559. doi: 10.3389/fpubh.2026.1790559 (PMC13617030; doi:10.3389/fpubh.2026.1790559)
Supplement: Supplementary file 1 [file Data_sheet_1.docx]

**Geospatial Modeling of Environmental and Socioeconomic Drivers of Health Outcomes**

Amirhooman Yalali, Hanadi S. Rifai, and Omolola E. Adepoju

**Supplementary Information**

Table S1. Statistical modeling results using ordinary least squares regression (2020)

| **Outcome** | **Statistic** | **Housing Age** | **Poverty Rate** | **Unemployment Rate** | **Uninsured Rate** | **Impervious Surface** | **Ozone** | **PM2.5** |
| --- | --- | --- | --- | --- | --- | --- | --- | --- |
| **Blood Pressure** | **Coefficient (p-value)** | 0.064*** (<0.001) | 0.215*** (<0.001) | 0.401*** (<0.001) | -0.243*** (<0.001) | -0.110*** (0.001) | -0.182 (0.316) | -0.196 (0.528) |
|  | **VIF** | 1.74 | 3.82 | 1.17 | 3.55 | 1.38 | 4.21 | 3.35 |
|  | **Adjusted R²** | 0.542 | | | | | | |
|  | **AICc** | 4526 | | | | | | |
| **Cancer** | **Coefficient (p-value)** | 0.024*** (0.001) | -0.017*** (0.003) | 0.013 (0.117) | -0.059*** (<0.001) | -0.016*** (0.004) | 0.110* (0.042) | -0.081 (0.372) |
|  | **VIF** | 1.75 | 3.87 | 1.18 | 3.5 | 1.4 | 4.18 | 3.33 |
|  | **Adjusted R²** | 0.551 | | | | | | |
|  | **AICc** | 2129 | | | | | | |
| **Asthma** | **Coefficient (p-value)** | 0.058*** (<0.001) | 0.079*** (<0.001) | 0.014 (0.096) | -0.028*** (0.001) | -0.012*** (0.003) | 0.061 (0.083) | 0.096 (0.241) |
|  | **VIF** | 1.73 | 3.8 | 1.16 | 3.52 | 1.39 | 4.25 | 3.38 |
|  | **Adjusted R²** | 0.718 | | | | | | |
|  | **AICc** | 1582 | | | | | | |
| **COPD** | **Coefficient (p-value)** | 0.021*** (0.002) | 0.070*** (<0.001) | 0.085*** (<0.001) | -0.041*** (<0.001) | -0.027*** (0.001) | 0.317*** (<0.001) | 0.283* (0.017) |
|  | **VIF** | 1.76 | 3.85 | 1.19 | 3.58 | 1.41 | 4.23 | 3.36 |
|  | **Adjusted R²** | 0.721 | | | | | | |
|  | **AICc** | 2254 | | | | | | |
| **Diabetes** | **Coefficient (p-value)** | 0.032*** (0.001) | 0.139*** (<0.001) | 0.198*** (<0.001) | -0.052*** (<0.001) | -0.060*** (0.002) | -0.010 (0.192) | 0.326 (0.233) |
|  | **VIF** | 1.78 | 3.89 | 1.2 | 3.57 | 1.42 | 4.27 | 3.4 |
|  | **Adjusted R²** | 0.728 | | | | | | |
|  | **AICc** | 3462 | | | | | | |
| **Mental Health** | **Coefficient (p-value)** | -0.006*** (<0.001) | 0.092*** (<0.001) | 0.058*** (<0.001) | 0.043*** (<0.001) | -0.004 (0.141) | 0.481*** (<0.001) | 0.605*** (<0.001) |
|  | **VIF** | 1.72 | 3.84 | 1.18 | 3.53 | 1.38 | 4.2 | 3.37 |
|  | **Adjusted R²** | 0.841 | | | | | | |
|  | **AICc** | 2203 | | | | | | |
| **Stroke** | **Coefficient (p-value)** | 0.010*** (0.001) | 0.045*** (<0.001) | 0.081*** (<0.001) | -0.038*** (<0.001) | -0.019*** (0.002) | -0.071 (0.065) | 0.017 (0.624) |
|  | **VIF** | 1.74 | 3.86 | 1.17 | 3.54 | 1.4 | 4.22 | 3.34 |
|  | **Adjusted R²** | 0.639 | | | | | | |
|  | **AICc** | 1741 | | | | | | |

Note: * indicates statistical significance at the 5% level (p < 0.05), ** at the 1% level (p < 0.01), and *** at the 0.1% level (p < 0.001).

Table S2. Statistical modeling results using ordinary least squares regression (2021)

| **Outcome** | **Statistic** | **Housing Age** | **Poverty Rate** | **Unemployment Rate** | **Uninsured Rate** | **Impervious Surface** | **Ozone** | **PM2.5** |
| --- | --- | --- | --- | --- | --- | --- | --- | --- |
| **Blood Pressure** | **Coefficient (p-value)** | 0.066*** (0.000) | 0.218*** (0.000) | 0.412*** (0.000) | -0.249*** (0.000) | -0.114*** (0.001) | -0.190 (0.382) | -0.202 (0.497) |
|  | **VIF** | 1.76 | 3.85 | 1.19 | 3.59 | 1.4 | 4.25 | 3.39 |
|  | **Adjusted R²** | 0.551 | | | | | | |
|  | **AICc** | 4487 | | | | | | |
| **Cancer** | **Coefficient (p-value)** | 0.025*** (0.001) | -0.019*** (0.002) | 0.012 (0.121) | -0.061*** (0.000) | -0.017*** (0.003) | 0.115* (0.038) | -0.085 (0.394) |
|  | **VIF** | 1.77 | 3.88 | 1.2 | 3.54 | 1.41 | 4.22 | 3.36 |
|  | **Adjusted R²** | 0.562 | | | | | | |
|  | **AICc** | 2087 | | | | | | |
| **Asthma** | **Coefficient (p-value)** | 0.060*** (0.000) | 0.081*** (0.000) | 0.013 (0.107) | -0.029*** (0.001) | -0.013*** (0.002) | 0.064 (0.071) | 0.099 (0.213) |
|  | **VIF** | 1.74 | 3.83 | 1.18 | 3.56 | 1.39 | 4.27 | 3.37 |
|  | **Adjusted R²** | 0.731 | | | | | | |
|  | **AICc** | 1503 | | | | | | |
| **COPD** | **Coefficient (p-value)** | 0.022*** (0.001) | 0.072*** (0.000) | 0.087*** (0.000) | -0.042*** (0.000) | -0.028*** (0.001) | 0.324*** (0.000) | 0.290* (0.015) |
|  | **VIF** | 1.78 | 3.86 | 1.21 | 3.6 | 1.42 | 4.24 | 3.38 |
|  | **Adjusted R²** | 0.735 | | | | | | |
|  | **AICc** | 2229 | | | | | | |
| **Diabetes** | **Coefficient (p-value)** | 0.033*** (0.001) | 0.143*** (0.000) | 0.203*** (0.000) | -0.053*** (0.000) | -0.061*** (0.002) | -0.011 (0.176) | 0.332 (0.221) |
|  | **VIF** | 1.79 | 3.9 | 1.22 | 3.58 | 1.43 | 4.28 | 3.41 |
|  | **Adjusted R²** | 0.736 | | | | | | |
|  | **AICc** | 3417 | | | | | | |
| **Mental Health** | **Coefficient (p-value)** | -0.007*** (0.000) | 0.094*** (0.000) | 0.060*** (0.000) | 0.044*** (0.000) | -0.004 (0.134) | 0.488*** (0.000) | 0.611*** (0.000) |
|  | **VIF** | 1.73 | 3.85 | 1.19 | 3.55 | 1.4 | 4.23 | 3.39 |
|  | **Adjusted R²** | 0.852 | | | | | | |
|  | **AICc** | 2175 | | | | | | |
| **Stroke** | **Coefficient (p-value)** | 0.010*** (0.001) | 0.046*** (0.000) | 0.082*** (0.000) | -0.039*** (0.000) | -0.020*** (0.002) | -0.073 (0.061) | 0.018 (0.602) |
|  | **VIF** | 1.75 | 3.87 | 1.18 | 3.56 | 1.41 | 4.26 | 3.36 |
|  | **Adjusted R²** | 0.645 | | | | | | |
|  | **AICc** | 1679 | | | | | | |

Note**:** * indicates statistical significance at the 5% level (p < 0.05), ** at the 1% level (p < 0.01), and *** at the 0.1% level (p < 0.001).

Table S3. Statistical modeling results using ordinary least squares regression (2023)

| **Outcome** | **Statistic** | **Housing Age** | **Poverty Rate** | **Unemployment Rate** | **Uninsured Rate** | **Impervious Surface** | **Ozone** | **PM2.5** |
| --- | --- | --- | --- | --- | --- | --- | --- | --- |
| **Blood Pressure** | **Coefficient (p-value)** | 0.071*** (0.000) | 0.227*** (0.000) | 0.431*** (0.000) | -0.255*** (0.000) | -0.119*** (0.001) | -0.188 (0.401) | -0.210 (0.486) |
|  | **VIF** | 1.81 | 3.93 | 1.22 | 3.63 | 1.43 | 4.31 | 3.43 |
|  | **Adjusted R²** | 0.568 | | | | | | |
|  | **AICc** | 4392 | | | | | | |
| **Cancer** | **Coefficient (p-value)** | 0.027*** (0.001) | -0.019*** (0.002) | 0.011 (0.142) | -0.064*** (0.000) | -0.018*** (0.002) | 0.119* (0.034) | -0.089 (0.362) |
|  | **VIF** | 1.8 | 3.9 | 1.21 | 3.59 | 1.44 | 4.28 | 3.4 |
|  | **Adjusted R²** | 0.576 | | | | | | |
|  | **AICc** | 2048 | | | | | | |
| **Asthma** | **Coefficient (p-value)** | 0.063*** (0.000) | 0.085*** (0.000) | 0.014 (0.102) | -0.031*** (0.001) | -0.014*** (0.001) | 0.067 (0.069) | 0.101 (0.208) |
|  | **VIF** | 1.79 | 3.89 | 1.2 | 3.6 | 1.42 | 4.33 | 3.44 |
|  | **Adjusted R²** | 0.746 | | | | | | |
|  | **AICc** | 1491 | | | | | | |
| **COPD** | **Coefficient (p-value)** | 0.023*** (0.001) | 0.075*** (0.000) | 0.090*** (0.000) | -0.043*** (0.000) | -0.029*** (0.001) | 0.330*** (0.000) | 0.297** (0.011) |
|  | **VIF** | 1.82 | 3.92 | 1.22 | 3.62 | 1.43 | 4.29 | 3.42 |
|  | **Adjusted R²** | 0.742 | | | | | | |
|  | **AICc** | 2205 | | | | | | |
| **Diabetes** | **Coefficient (p-value)** | 0.036*** (0.001) | 0.150*** (0.000) | 0.210*** (0.000) | -0.055*** (0.000) | -0.064*** (0.001) | -0.012 (0.158) | 0.338 (0.207) |
|  | **VIF** | 1.83 | 3.94 | 1.21 | 3.61 | 1.44 | 4.35 | 3.46 |
|  | **Adjusted R²** | 0.753 | | | | | | |
|  | **AICc** | 3339 | | | | | | |
| **Mental Health** | **Coefficient (p-value)** | -0.007*** (0.000) | 0.096*** (0.000) | 0.062*** (0.000) | 0.046*** (0.000) | -0.005 (0.121) | 0.492*** (0.000) | 0.615*** (0.000) |
|  | **VIF** | 1.8 | 3.91 | 1.2 | 3.6 | 1.42 | 4.3 | 3.41 |
|  | **Adjusted R²** | 0.861 | | | | | | |
|  | **AICc** | 2124 | | | | | | |
| **Stroke** | **Coefficient (p-value)** | 0.011*** (0.001) | 0.047*** (0.000) | 0.085*** (0.000) | -0.041*** (0.000) | -0.021*** (0.001) | -0.075 (0.058) | 0.016 (0.588) |
|  | **VIF** | 1.81 | 3.93 | 1.21 | 3.61 | 1.43 | 4.31 | 3.43 |
|  | **Adjusted R²** | 0.659 | | | | | | |
|  | **AICc** | 1713 | | | | | | |

Note: * indicates statistical significance at the 5% level (p < 0.05), ** at the 1% level (p < 0.01), and *** at the 0.1% level (p < 0.001).

Table S4. Statistical modeling results using ordinary least squares regression (2024)

| **Outcome** | **Statistic** | **Housing Age** | **Poverty Rate** | **Unemployment Rate** | **Uninsured Rate** | **Impervious Surface** | **Ozone** | **PM2.5** |
| --- | --- | --- | --- | --- | --- | --- | --- | --- |
| **Blood Pressure** | **Coefficient (p-value)** | 0.068*** (0.000) | 0.223*** (0.000) | 0.419*** (0.000) | -0.250*** (0.000) | -0.116*** (0.001) | -0.192 (0.369) | -0.204 (0.508) |
|  | **VIF** | 1.8 | 3.92 | 1.21 | 3.61 | 1.42 | 4.28 | 3.41 |
|  | **Adjusted R²** | 0.559 | | | | | | |
|  | **AICc** | 4441 | | | | | | |
| **Cancer** | **Coefficient (p-value)** | 0.026*** (0.001) | -0.018*** (0.002) | 0.013 (0.127) | -0.062*** (0.000) | -0.017*** (0.002) | 0.117* (0.037) | -0.086 (0.384) |
|  | **VIF** | 1.79 | 3.91 | 1.2 | 3.57 | 1.43 | 4.25 | 3.38 |
|  | **Adjusted R²** | 0.569 | | | | | | |
|  | **AICc** | 2067 | | | | | | |
| **Asthma** | **Coefficient (p-value)** | 0.062*** (0.000) | 0.084*** (0.000) | 0.013 (0.112) | -0.030*** (0.001) | -0.013*** (0.002) | 0.065 (0.076) | 0.100 (0.229) |
|  | **VIF** | 1.78 | 3.89 | 1.19 | 3.58 | 1.41 | 4.31 | 3.43 |
|  | **Adjusted R²** | 0.737 | | | | | | |
|  | **AICc** | 1517 | | | | | | |
| **COPD** | **Coefficient (p-value)** | 0.022*** (0.001) | 0.073*** (0.000) | 0.088*** (0.000) | -0.042*** (0.000) | -0.028*** (0.001) | 0.327*** (0.000) | 0.292* (0.013) |
|  | **VIF** | 1.81 | 3.93 | 1.21 | 3.6 | 1.42 | 4.3 | 3.41 |
|  | **Adjusted R²** | 0.739 | | | | | | |
|  | **AICc** | 2216 | | | | | | |
| **Diabetes** | **Coefficient (p-value)** | 0.034*** (0.001) | 0.145*** (0.000) | 0.207*** (0.000) | -0.054*** (0.000) | -0.062*** (0.002) | -0.011 (0.183) | 0.335 (0.216) |
|  | **VIF** | 1.82 | 3.94 | 1.21 | 3.59 | 1.43 | 4.33 | 3.45 |
|  | **Adjusted R²** | 0.742 | | | | | | |
|  | **AICc** | 3381 | | | | | | |
| **Mental Health** | **Coefficient (p-value)** | -0.007*** (0.000) | 0.095*** (0.000) | 0.061*** (0.000) | 0.045*** (0.000) | -0.004 (0.129) | 0.490*** (0.000) | 0.613*** (0.000) |
|  | **VIF** | 1.79 | 3.9 | 1.2 | 3.58 | 1.42 | 4.29 | 3.4 |
|  | **Adjusted R²** | 0.857 | | | | | | |
|  | **AICc** | 2151 | | | | | | |
| **Stroke** | **Coefficient (p-value)** | 0.011*** (0.001) | 0.047*** (0.000) | 0.084*** (0.000) | -0.040*** (0.000) | -0.020*** (0.001) | -0.074 (0.063) | 0.017 (0.606) |
|  | **VIF** | 1.8 | 3.92 | 1.2 | 3.59 | 1.42 | 4.32 | 3.42 |
|  | **Adjusted R²** | 0.652 | | | | | | |
|  | **AICc** | 1698 | | | | | | |

Note: * indicates statistical significance at the 5% level (p < 0.05), ** at the 1% level (p < 0.01), and *** at the 0.1% level (p < 0.001).

Table S5. Statistical modeling results using GWR (2020)

| **Outcome** | **Statistic** | **Housing Age** | **Poverty Rate** | **Unemployment Rate** | **Uninsured Rate** | **Impervious Surface** | **Ozone** | **PM2.5** |
| --- | --- | --- | --- | --- | --- | --- | --- | --- |
| **Blood Pressure** | **Mean** | 0.219 | 0.482 | 0.128 | -0.335 | -0.222 | -0.111 | 0.118 |
|  | **Median** | 0.210 | 0.448 | 0.093 | -0.295 | -0.180 | -0.276 | 0.112 |
|  | **SD** | 0.185 | 0.220 | 0.159 | 0.002 | 0.202 | 0.354 | 0.500 |
|  | **Adjusted R²** | 0.693 | | | | | | |
|  | **AICc** | 2748.7097 | | | | | | |
| **Cancer** | **Mean** | 0.433 | -0.131 | 0.029 | -0.466 | -0.173 | 0.560 | 0.331 |
|  | **Median** | 0.402 | -0.141 | 0.030 | -0.448 | -0.145 | 0.516 | 0.225 |
|  | **SD** | 0.221 | 0.170 | 0.003 | 0.134 | 0.240 | 0.049 | 0.606 |
|  | **Adjusted R²** | 0.683 | | | | | | |
|  | **AICc** | 1764.5525 | | | | | | |
| **Asthma** | **Mean** | -0.005 | 0.671 | 0.143 | -0.049 | -0.064 | -0.203 | -0.058 |
|  | **Median** | -0.009 | 0.689 | 0.175 | -0.050 | -0.031 | -0.036 | 0.018 |
|  | **SD** | 0.088 | 0.217 | 0.113 | 0.074 | 0.167 | 0.639 | 0.706 |
|  | **Adjusted R²** | 0.786 | | | | | | |
|  | **AICc** | 1321.1 | | | | | | |
| **COPD** | **Mean** | 0.281 | 0.686 | 0.091 | -0.177 | -0.109 | 0.493 | 0.532 |
|  | **Median** | 0.289 | 0.637 | 0.068 | -0.172 | -0.162 | 0.559 | 0.503 |
|  | **SD** | 0.013 | 0.218 | 0.108 | 0.007 | 0.209 | 0.102 | 0.282 |
|  | **Adjusted R²** | 0.789 | | | | | | |
|  | **AICc** | 1731.5303 | | | | | | |
| **Diabetes** | **Mean** | 0.141 | 0.451 | 0.070 | -0.070 | -0.139 | 0.032 | 0.495 |
|  | **Median** | 0.156 | 0.471 | 0.064 | -0.066 | -0.118 | 0.034 | 0.627 |
|  | **SD** | 0.152 | 0.294 | 0.072 | 0.082 | 0.151 | 0.139 | 0.542 |
|  | **Adjusted R²** | 0.827 | | | | | | |
|  | **AICc** | 1586 | | | | | | |
| **Mental Health** | **Mean** | 0.063 | 0.634 | 0.037 | 0.205 | 0.017 | 0.151 | 0.100 |
|  | **Median** | 0.053 | 0.705 | 0.030 | 0.222 | 0.019 | 0.152 | 0.199 |
|  | **SD** | 0.057 | 0.111 | 0.047 | 0.146 | 0.029 | 0.084 | 0.401 |
|  | **Adjusted R²** | 0.872 | | | | | | |
|  | **AICc** | 983.5993 | | | | | | |
| **Stroke** | **Mean** | 0.190 | 0.538 | 0.120 | -0.205 | -0.140 | 0.275 | 0.708 |
|  | **Median** | 0.188 | 0.432 | 0.085 | -0.187 | -0.115 | 0.302 | 0.666 |
|  | **SD** | 0.003 | 0.303 | 0.170 | 0.036 | 0.173 | 0.252 | 0.356 |
|  | **Adjusted R²** | 0.743 | | | | | | |
|  | **AICc** | 1328 | | | | | | |

Table S6. Statistical modeling results using GWR (2021)

| **Outcome** | **Statistic** | **Housing Age** | **Poverty Rate** | **Unemployment Rate** | **Uninsured Rate** | **Impervious Surface** | **Ozone** | **PM2.5** |
| --- | --- | --- | --- | --- | --- | --- | --- | --- |
| **Blood Pressure** | **Mean** | 0.224 | 0.488 | 0.136 | -0.293 | -0.276 | -0.118 | 0.098 |
|  | **Median** | 0.183 | 0.479 | 0.088 | -0.321 | -0.193 | -0.270 | 0.122 |
|  | **SD** | 0.187 | 0.222 | 0.172 | 0.002 | 0.198 | 0.379 | 0.478 |
|  | **Adjusted R²** | 0.693 | | | | | | |
|  | **AICc** | 2297 | | | | | | |
| **Cancer** | **Mean** | 0.441 | -0.151 | 0.031 | -0.530 | -0.157 | 0.544 | 0.300 |
|  | **Median** | 0.417 | -0.158 | 0.036 | -0.512 | -0.154 | 0.642 | 0.260 |
|  | **SD** | 0.216 | 0.170 | 0.003 | 0.133 | 0.238 | 0.053 | 0.688 |
|  | **Adjusted R²** | 0.634 | | | | | | |
|  | **AICc** | 1662 | | | | | | |
| **Asthma** | **Mean** | -0.005 | 0.696 | 0.138 | -0.044 | -0.063 | -0.178 | -0.064 |
|  | **Median** | -0.009 | 0.641 | 0.189 | -0.054 | -0.027 | -0.039 | 0.018 |
|  | **SD** | 0.088 | 0.198 | 0.113 | 0.072 | 0.170 | 0.623 | 0.685 |
|  | **Adjusted R²** | 0.771 | | | | | | |
|  | **AICc** | 1192.1 | | | | | | |
| **COPD** | **Mean** | 0.237 | 0.731 | 0.109 | -0.191 | -0.113 | 0.482 | 0.570 |
|  | **Median** | 0.248 | 0.683 | 0.076 | -0.167 | -0.151 | 0.419 | 0.485 |
|  | **SD** | 0.013 | 0.206 | 0.111 | 0.007 | 0.208 | 0.104 | 0.319 |
|  | **Adjusted R²** | 0.741 | | | | | | |
|  | **AICc** | 1425.5303 | | | | | | |
| **Diabetes** | **Mean** | 0.165 | 0.474 | 0.069 | -0.076 | -0.136 | 0.031 | 0.453 |
|  | **Median** | 0.172 | 0.421 | 0.075 | -0.066 | -0.117 | 0.036 | 0.543 |
|  | **SD** | 0.150 | 0.287 | 0.071 | 0.085 | 0.149 | 0.137 | 0.508 |
|  | **Adjusted R²** | 0.792 | | | | | | |
|  | **AICc** | 1723 | | | | | | |
| **Mental Health** | **Mean** | 0.050 | 0.657 | 0.041 | 0.214 | 0.018 | 0.174 | 0.109 |
|  | **Median** | 0.055 | 0.708 | 0.031 | 0.196 | 0.021 | 0.165 | 0.199 |
|  | **SD** | 0.064 | 0.110 | 0.045 | 0.155 | 0.029 | 0.082 | 0.389 |
|  | **Adjusted R²** | 0.872 | | | | | | |
|  | **AICc** | 1239.5993 | | | | | | |
| **Stroke** | **Mean** | 0.168 | 0.572 | 0.135 | -0.211 | -0.144 | 0.291 | 0.572 |
|  | **Median** | 0.183 | 0.449 | 0.087 | -0.199 | -0.102 | 0.272 | 0.729 |
|  | **SD** | 0.002 | 0.287 | 0.166 | 0.034 | 0.171 | 0.255 | 0.374 |
|  | **Adjusted R²** | 0.703 | | | | | | |
|  | **AICc** | 1412 | | | | | | |

Table S7. Statistical modeling results using GWR (2023)

| **Outcome** | **Statistic** | **Housing Age** | **Poverty Rate** | **Unemployment Rate** | **Uninsured Rate** | **Impervious Surface** | **Ozone** | **PM2.5** |
| --- | --- | --- | --- | --- | --- | --- | --- | --- |
| **Blood Pressure** | **Mean** | 0.191 | 0.448 | 0.122 | -0.303 | -0.216 | -0.116 | 0.096 |
|  | **Median** | 0.198 | 0.438 | 0.086 | -0.306 | -0.179 | -0.266 | 0.136 |
|  | **SD** | 0.185 | 0.222 | 0.149 | 0.002 | 0.212 | 0.317 | 0.430 |
|  | **Adjusted R²** | 0.679 | | | | | | |
|  | **AICc** | 3581 | | | | | | |
| **Cancer** | **Mean** | 0.431 | -0.143 | 0.030 | -0.495 | -0.180 | 0.576 | 0.281 |
|  | **Median** | 0.394 | -0.155 | 0.028 | -0.498 | -0.156 | 0.625 | 0.245 |
|  | **SD** | 0.219 | 0.153 | 0.002 | 0.122 | 0.211 | 0.049 | 0.594 |
|  | **Adjusted R²** | 0.683 | | | | | | |
|  | **AICc** | 1872 | | | | | | |
| **Asthma** | **Mean** | -0.004 | 0.700 | 0.157 | -0.044 | -0.063 | -0.192 | -0.061 |
|  | **Median** | -0.009 | 0.742 | 0.165 | -0.052 | -0.031 | -0.037 | 0.020 |
|  | **SD** | 0.077 | 0.195 | 0.105 | 0.069 | 0.158 | 0.652 | 0.644 |
|  | **Adjusted R²** | 0.764 | | | | | | |
|  | **AICc** | 1207.1 | | | | | | |
| **COPD** | **Mean** | 0.274 | 0.650 | 0.097 | -0.188 | -0.132 | 0.549 | 0.497 |
|  | **Median** | 0.246 | 0.609 | 0.077 | -0.184 | -0.151 | 0.553 | 0.497 |
|  | **SD** | 0.014 | 0.207 | 0.099 | 0.007 | 0.205 | 0.099 | 0.275 |
|  | **Adjusted R²** | 0.791 | | | | | | |
|  | **AICc** | 1587.5303 | | | | | | |
| **Diabetes** | **Mean** | 0.155 | 0.444 | 0.072 | -0.075 | -0.149 | 0.028 | 0.463 |
|  | **Median** | 0.153 | 0.453 | 0.062 | -0.071 | -0.123 | 0.034 | 0.501 |
|  | **SD** | 0.137 | 0.271 | 0.074 | 0.081 | 0.127 | 0.127 | 0.491 |
|  | **Adjusted R²** | 0.771 | | | | | | |
|  | **AICc** | 1919 | | | | | | |
| **Mental Health** | **Mean** | 0.058 | 0.727 | 0.038 | 0.192 | 0.021 | 0.173 | 0.099 |
|  | **Median** | 0.057 | 0.687 | 0.031 | 0.194 | 0.019 | 0.156 | 0.184 |
|  | **SD** | 0.055 | 0.104 | 0.041 | 0.159 | 0.024 | 0.082 | 0.388 |
|  | **Adjusted R²** | 0.823 | | | | | | |
|  | **AICc** | 1124 | | | | | | |
| **Stroke** | **Mean** | 0.179 | 0.495 | 0.138 | -0.211 | -0.146 | 0.302 | 0.581 |
|  | **Median** | 0.178 | 0.482 | 0.077 | -0.207 | -0.108 | 0.274 | 0.731 |
|  | **SD** | 0.002 | 0.278 | 0.164 | 0.030 | 0.170 | 0.254 | 0.346 |
|  | **Adjusted R²** | 0.7109 | | | | | | |
|  | **AICc** | 1392 | | | | | | |

Table S8. Statistical modeling results using GWR (2024)

| **Outcome** | **Statistic** | **Housing Age** | **Poverty Rate** | **Unemployment Rate** | **Uninsured Rate** | **Impervious Surface** | **Ozone** | **PM2.5** |
| --- | --- | --- | --- | --- | --- | --- | --- | --- |
| **Blood Pressure** | **Mean** | 0.205 | 0.513 | 0.117 | -0.278 | -0.209 | -0.116 | 0.098 |
|  | **Median** | 0.214 | 0.420 | 0.096 | -0.308 | -0.185 | -0.264 | 0.133 |
|  | **SD** | 0.189 | 0.225 | 0.158 | 0.002 | 0.196 | 0.356 | 0.453 |
|  | **Adjusted R²** | 0.631 | | | | | | |
|  | **AICc** | 3012 | | | | | | |
| **Cancer** | **Mean** | 0.425 | -0.144 | 0.029 | -0.476 | -0.170 | 0.594 | 0.274 |
|  | **Median** | 0.410 | -0.140 | 0.029 | -0.502 | -0.144 | 0.628 | 0.267 |
|  | **SD** | 0.217 | 0.143 | 0.003 | 0.124 | 0.231 | 0.051 | 0.574 |
|  | **Adjusted R²** | 0.649 | | | | | | |
|  | **AICc** | 1422 | | | | | | |
| **Asthma** | **Mean** | -0.005 | 0.645 | 0.144 | -0.050 | -0.057 | -0.214 | -0.064 |
|  | **Median** | -0.009 | 0.647 | 0.169 | -0.050 | -0.032 | -0.043 | 0.019 |
|  | **SD** | 0.085 | 0.188 | 0.107 | 0.073 | 0.151 | 0.674 | 0.680 |
|  | **Adjusted R²** | 0.782 | | | | | | |
|  | **AICc** | 1423 | | | | | | |
| **COPD** | **Mean** | 0.243 | 0.656 | 0.092 | -0.175 | -0.133 | 0.506 | 0.483 |
|  | **Median** | 0.265 | 0.680 | 0.074 | -0.187 | -0.157 | 0.557 | 0.521 |
|  | **SD** | 0.011 | 0.210 | 0.099 | 0.007 | 0.189 | 0.093 | 0.299 |
|  | **Adjusted R²** | 0.742 | | | | | | |
|  | **AICc** | 1421.5303 | | | | | | |
| **Diabetes** | **Mean** | 0.167 | 0.455 | 0.072 | -0.073 | -0.138 | 0.031 | 0.435 |
|  | **Median** | 0.151 | 0.488 | 0.062 | -0.076 | -0.116 | 0.036 | 0.537 |
|  | **SD** | 0.153 | 0.256 | 0.069 | 0.086 | 0.137 | 0.134 | 0.485 |
|  | **Adjusted R²** | 0.735 | | | | | | |
|  | **AICc** | 2156 | | | | | | |
| **Mental Health** | **Mean** | 0.057 | 0.742 | 0.038 | 0.199 | 0.018 | 0.161 | 0.095 |
|  | **Median** | 0.055 | 0.658 | 0.032 | 0.202 | 0.021 | 0.149 | 0.197 |
|  | **SD** | 0.057 | 0.104 | 0.043 | 0.141 | 0.028 | 0.079 | 0.376 |
|  | **Adjusted R²** | 0.793 | | | | | | |
|  | **AICc** | 1396 | | | | | | |
| **Stroke** | **Mean** | 0.181 | 0.509 | 0.122 | -0.213 | -0.147 | 0.283 | 0.590 |
|  | **Median** | 0.186 | 0.460 | 0.083 | -0.203 | -0.101 | 0.298 | 0.765 |
|  | **SD** | 0.002 | 0.277 | 0.158 | 0.032 | 0.170 | 0.270 | 0.348 |
|  | **Adjusted R²** | 0.73 | | | | | | |
|  | **AICc** | 1128 | | | | | | |

Table S9. Statistical analysis results of MGWR modeling 2020

| **Outcome** | **Statistic** | **Housing Age** | **Poverty Rate** | **Unemployment Rate** | **Uninsured Rate** | **Impervious Surface** | **Ozone** | **PM2.5** |
| --- | --- | --- | --- | --- | --- | --- | --- | --- |
| **Blood Pressure** | **Mean** | 0.2355 | 0.5041 | 0.1323 | -0.3319 | -0.2339 | -0.1401 | 0.1181 |
|  | **Median** | 0.2268 | 0.4978 | 0.1048 | -0.3406 | -0.1989 | -0.3097 | 0.1383 |
|  | **SD** | 0.1497 | 0.1636 | 0.1232 |  | 0.1625 | 0.2914 | 0.3571 |
|  | **Adjusted R²** | 0.7888 | | | | | | |
|  | **AICc** | 1164.0992 | | | | | | |
| **Cancer** | **Mean** | 0.456 | -0.16 | 0.033 | -0.5435 | -0.1959 | 0.6524 | 0.3142 |
|  | **Median** | 0.4372 | -0.1621 | 0.0314 | -0.5385 | -0.1618 | 0.6235 | 0.2601 |
|  | **SD** | 0.1633 | 0.1285 | 0.0011 | 0.9635 | 0.1844 | 0.3232 | 0.4902 |
|  | **Adjusted R²** | 0.7281 | | | | | | |
|  | **AICc** | 1311.9884 | | | | | | |
| **Asthma** | **Mean** | 0.0029 | 0.7573 | 0.1623 | -0.052 | -0.07 | -0.2108 | -0.0835 |
|  | **Median** | -0.001 | 0.7051 | 0.1762 | -0.0646 | -0.044 | -0.0488 | 0.0345 |
|  | **SD** | 0.0631 | 0.1512 | 0.0867 | 0.0524 | 0.1204 | 0.5049 | 0.5557 |
|  | **Adjusted R²** | 0.8668 | | | | | | |
|  | **AICc** | 848.9004 | | | | | | |
| **COPD** | **Mean** | 0.307 | 0.7397 | 0.0998 | -0.1916 | -0.1471 | 0.5459 | 0.5569 |
|  | **Median** | 0.3034 | 0.7337 | 0.0814 | -0.1982 | -0.1568 | 0.5889 | 0.5735 |
|  | **SD** | 0.0095 | 0.1557 | 0.0743 | 0.0046 | 0.1605 | 0.0761 | 0.2211 |
|  | **Adjusted R²** | 0.8606 | | | | | | |
|  | **AICc** | 948.2476 | | | | | | |
| **Diabetes** | **Mean** | 0.1796 | 0.5099 | 0.0842 | -0.0803 | -0.1516 | 0.041 | 0.4795 |
|  | **Median** | 0.1735 | 0.4954 | 0.0729 | -0.0806 | -0.1322 | 0.0407 | 0.5978 |
|  | **SD** | 0.1183 | 0.2262 | 0.0575 | 0.0659 | 0.1085 | 0.0974 | 0.3956 |
|  | **Adjusted R²** | 0.892 | | | | | | |
|  | **AICc** | 704.1595 | | | | | | |
| **Mental Health** | **Mean** | 0.0629 | 0.7492 | 0.0452 | 0.229 | 0.0212 | 0.1795 | 0.1221 |
|  | **Median** | 0.0689 | 0.712 | 0.0346 | 0.2315 | 0.0204 | 0.1686 | 0.1976 |
|  | **SD** | 0.0431 | 0.0837 | 0.0319 | 0.1147 | 0.0217 | 0.0595 | 0.3136 |
|  | **Adjusted R²** | 0.8782 | | | | | | |
|  | **AICc** | 436.4826 | | | | | | |
| **Stroke** | **Mean** | 0.207 | 0.5668 | 0.1439 | -0.2235 | -0.1476 | 0.3214 | 0.6691 |
|  | **Median** | 0.2036 | 0.5161 | 0.019 | -0.2095 | -0.1374 | 0.3771 | 0.7501 |
|  | **SD** | <0.0016 | 0.2187 | 0.1251 | 0.0241 | 0.1374 | 0.1922 | 0.2868 |
|  | **Adjusted R²** | 0.8366 | | | | | | |
|  | **AICc** | 984.4495 | | | | | | |

Table S10. Statistical analysis results of MGWR modeling 2021

| **Outcome** | **Statistic** | **Housing Age** | **Poverty Rate** | **Unemployment Rate** | **Uninsured Rate** | **Impervious Surface** | **Ozone** | **PM2.5** |
| --- | --- | --- | --- | --- | --- | --- | --- | --- |
| **Blood Pressure** | **Mean** | 0.2399 | 0.5225 | 0.1357 | -0.3263 | -0.2333 | -0.1328 | 0.1163 |
|  | **Median** | 0.229 | 0.4946 | 0.0964 | -0.3453 | -0.2 | -0.3045 | 0.1512 |
|  | **SD** | 0.1404 | 0.1701 | 0.121 | <0.0016 | 0.154 | 0.2739 | 0.3562 |
|  | **Adjusted R²** | 0.7278 | | | | | | |
|  | **AICc** | 1213.0192 | | | | | | |
| **Cancer** | **Mean** | 0.4624 | -0.1537 | 0.0337 | -0.5021 | -0.1918 | 0.5947 | 0.3167 |
|  | **Median** | 0.4116 | -0.1625 | 0.0332 | -0.5046 | -0.157 | 0.5915 | 0.2613 |
|  | **SD** | 0.1729 | 0.1286 | 0.0011 | 1.0232 | 0.1732 | 0.3163 | 0.4892 |
|  | **Adjusted R²** | 0.6914 | | | | | | |
|  | **AICc** | 1320.8555 | | | | | | |
| **Asthma** | **Mean** | 0.0029 | 0.6998 | 0.1584 | -0.0521 | -0.0753 | -0.1994 | -0.089 |
|  | **Median** | -0.001 | 0.7244 | 0.1832 | -0.0639 | -0.0441 | -0.0519 | 0.0374 |
|  | **SD** | 0.0623 | 0.1578 | 0.0817 | 0.0558 | 0.1291 | 0.5034 | 0.5616 |
|  | **Adjusted R²** | 0.8869 | | | | | | |
|  | **AICc** | 799.1956 | | | | | | |
| **COPD** | **Mean** | 0.2955 | 0.6913 | 0.0994 | -0.2008 | -0.1413 | 0.5465 | 0.5669 |
|  | **Median** | 0.2778 | 0.6804 | 0.0864 | -0.1936 | -0.156 | 0.5543 | 0.5582 |
|  | **SD** | 0.009 | 0.1653 | 0.0786 | 0.0044 | 0.1713 | 0.0755 | 0.24 |
|  | **Adjusted R²** | 0.8015 | | | | | | |
|  | **AICc** | 983.7181 | | | | | | |
| **Diabetes** | **Mean** | 0.1882 | 0.5237 | 0.0782 | -0.0836 | -0.161 | 0.0386 | 0.4561 |
|  | **Median** | 0.1761 | 0.4946 | 0.0715 | -0.0832 | -0.1344 | 0.0391 | 0.5523 |
|  | **SD** | 0.1199 | 0.2283 | 0.0573 | 0.0684 | 0.1115 | 0.0951 | 0.4279 |
|  | **Adjusted R²** | 0.8462 | | | | | | |
|  | **AICc** | 723.5092 | | | | | | |
| **Mental Health** | **Mean** | 0.0592 | 0.7482 | 0.0433 | 0.2237 | 0.0212 | 0.1824 | 0.1175 |
|  | **Median** | 0.0678 | 0.6882 | 0.0375 | 0.2117 | 0.0194 | 0.1772 | 0.2004 |
|  | **SD** | 0.045 | 0.0793 | 0.0321 | 0.118 | 0.0206 | 0.0644 | 0.2891 |
|  | **Adjusted R²** | 0.9425 | | | | | | |
|  | **AICc** | 445.5694 | | | | | | |
| **Stroke** | **Mean** | 0.2119 | 0.5581 | 0.1369 | -0.2207 | -0.1598 | 0.3253 | 0.6803 |
|  | **Median** | 0.212 | 0.4852 | 0.0197 | -0.2173 | -0.132 | 0.3918 | 0.7926 |
|  | **SD** | <0.0016 | 0.2318 | 0.126 | 0.0231 | 0.1322 | 0.1993 | 0.2996 |
|  | **Adjusted R²** | 0.8247 | | | | | | |
|  | **AICc** | 1010.1987 | | | | | | |

Table S11. Statistical analysis results of MGWR modeling 2023

| **Outcome** | **Statistic** | **Housing Age** | **Poverty Rate** | **Unemployment Rate** | **Uninsured Rate** | **Impervious Surface** | **Ozone** | **PM2.5** |
| --- | --- | --- | --- | --- | --- | --- | --- | --- |
| **Blood Pressure** | **Mean** | 0.2342 | 0.5199 | 0.1337 | -0.3487 | -0.2373 | -0.1414 | 0.1191 |
|  | **Median** | 0.2275 | 0.5234 | 0.1046 | -0.3321 | -0.2082 | -0.3063 | 0.1462 |
|  | **SD** | 0.1519 | 0.1686 | 0.1224 | <0.0016 | 0.157 | 0.2939 | 0.3647 |
|  | **Adjusted R²** | 0.7626 | | | | | | |
|  | **AICc** | 1202.4894 | | | | | | |
| **Cancer** | **Mean** | 0.4355 | -0.1531 | 0.0319 | -0.5236 | -0.1985 | 0.6492 | 0.3265 |
|  | **Median** | 0.4301 | -0.1676 | 0.0318 | -0.525 | -0.1633 | 0.6169 | 0.2726 |
|  | **SD** | 0.1617 | 0.1196 | 0.0011 | 1.0065 | 0.1774 | 0.3125 | 0.496 |
|  | **Adjusted R²** | 0.6998 | | | | | | |
|  | **AICc** | 1417.726 | | | | | | |
| **Asthma** | **Mean** | 0.0031 | 0.717 | 0.1582 | -0.0524 | -0.0758 | -0.2018 | -0.0898 |
|  | **Median** | -0.001 | 0.7262 | 0.1692 | -0.0642 | -0.0441 | -0.0513 | 0.0344 |
|  | **SD** | 0.0589 | 0.1515 | 0.084 | 0.0559 | 0.1233 | 0.5009 | 0.5715 |
|  | **Adjusted R²** | 0.8151 | | | | | | |
|  | **AICc** | 812.6824 | | | | | | |
| **COPD** | **Mean** | 0.2912 | 0.6939 | 0.1031 | -0.194 | -0.1422 | 0.5178 | 0.562 |
|  | **Median** | 0.2825 | 0.7011 | 0.0843 | -0.1981 | -0.1574 | 0.5514 | 0.5355 |
|  | **SD** | 0.009 | 0.1599 | 0.0795 | 0.0048 | 0.159 | 0.0768 | 0.2333 |
|  | **Adjusted R²** | 0.7977 | | | | | | |
|  | **AICc** | 909.669 | | | | | | |
| **Diabetes** | **Mean** | 0.1765 | 0.4912 | 0.0822 | -0.0837 | -0.1623 | 0.04 | 0.4644 |
|  | **Median** | 0.1873 | 0.4848 | 0.0757 | -0.0817 | -0.1384 | 0.039 | 0.5811 |
|  | **SD** | 0.1195 | 0.2133 | 0.0576 | 0.0659 | 0.1128 | 0.0994 | 0.4359 |
|  | **Adjusted R²** | 0.8662 | | | | | | |
|  | **AICc** | 711.0246 | | | | | | |
| **Mental Health** | **Mean** | 0.063 | 0.7191 | 0.0473 | 0.237 | 0.022 | 0.1818 | 0.1123 |
|  | **Median** | 0.069 | 0.7193 | 0.0375 | 0.2134 | 0.0207 | 0.172 | 0.2159 |
|  | **SD** | 0.0463 | 0.0792 | 0.0313 | 0.122 | 0.0202 | 0.0597 | 0.3054 |
|  | **Adjusted R²** | 0.9515 | | | | | | |
|  | **AICc** | 414.5014 | | | | | | |
| **Stroke** | **Mean** | 0.2143 | 0.5413 | 0.1456 | -0.2253 | -0.1567 | 0.3181 | 0.6526 |
|  | **Median** | 0.2086 | 0.5308 | 0.0186 | -0.2225 | -0.1329 | 0.4069 | 0.7874 |
|  | **SD** | <0.0016 | 0.2185 | 0.1265 | 0.0229 | 0.1296 | 0.2005 | 0.2809 |
|  | **Adjusted R²** | 0.8478 | | | | | | |
|  | **AICc** | 991.2276 | | | | | | |

Table S12. Statistical analysis results of MGWR modeling 2024

| **Outcome** | **Statistic** | **Housing Age** | **Poverty Rate** | **Unemployment Rate** | **Uninsured Rate** | **Impervious Surface** | **Ozone** | **PM2.5** |
| --- | --- | --- | --- | --- | --- | --- | --- | --- |
| **Blood Pressure** | **Mean** | 0.2344 | 0.524 | 0.1429 | -0.3461 | -0.2335 | -0.1363 | 0.1222 |
|  | **Median** | 0.2312 | 0.4822 | 0.0966 | -0.3166 | -0.207 | -0.2964 | 0.138 |
|  | **SD** | 0.1514 | 0.1781 | 0.1197 | <0.0016 | 0.1604 | 0.2941 | 0.3688 |
|  | **Adjusted R²** | 0.773 | | | | | | |
|  | **AICc** | 1233.9667 | | | | | | |
| **Cancer** | **Mean** | 0.4494 | -0.1597 | 0.0336 | -0.5144 | -0.1985 | 0.6267 | 0.3055 |
|  | **Median** | 0.4186 | -0.1669 | 0.0314 | -0.5302 | -0.1656 | 0.6153 | 0.2666 |
|  | **SD** | 0.1637 | 0.1303 | 0.0011 | 1.0412 | 0.1728 | 0.3046 | 0.4949 |
|  | **Adjusted R²** | 0.7259 | | | | | | |
|  | **AICc** | 1333.0618 | | | | | | |
| **Asthma** | **Mean** | 0.0029 | 0.6971 | 0.1698 | -0.0556 | -0.0729 | -0.2016 | -0.0851 |
|  | **Median** | -0.001 | 0.7275 | 0.1769 | -0.0653 | -0.0447 | -0.049 | 0.036 |
|  | **SD** | 0.0633 | 0.1457 | 0.0836 | 0.052 | 0.1219 | 0.5159 | 0.5735 |
|  | **Adjusted R²** | 0.8593 | | | | | | |
|  | **AICc** | 799.053 | | | | | | |
| **COPD** | **Mean** | 0.293 | 0.7231 | 0.1071 | -0.1996 | -0.1403 | 0.5498 | 0.5579 |
|  | **Median** | 0.2998 | 0.6904 | 0.0881 | -0.1867 | -0.1615 | 0.5735 | 0.5829 |
|  | **SD** | 0.0092 | 0.1573 | 0.0783 | 0.0047 | 0.1639 | 0.0763 | 0.2352 |
|  | **Adjusted R²** | 0.8557 | | | | | | |
|  | **AICc** | 940.5414 | | | | | | |
| **Diabetes** | **Mean** | 0.1721 | 0.5263 | 0.0791 | -0.082 | -0.1593 | 0.0388 | 0.4709 |
|  | **Median** | 0.1711 | 0.5021 | 0.0706 | -0.0867 | -0.127 | 0.04 | 0.5913 |
|  | **SD** | 0.119 | 0.2186 | 0.0541 | 0.066 | 0.1117 | 0.0956 | 0.4202 |
|  | **Adjusted R²** | 0.8582 | | | | | | |
|  | **AICc** | 718.2931 | | | | | | |
| **Mental Health** | **Mean** | 0.0617 | 0.7184 | 0.0451 | 0.2287 | 0.0217 | 0.179 | 0.1186 |
|  | **Median** | 0.0671 | 0.7512 | 0.0372 | 0.231 | 0.0209 | 0.1632 | 0.2151 |
|  | **SD** | 0.0454 | 0.0786 | 0.033 | 0.1148 | 0.0211 | 0.0607 | 0.3106 |
|  | **Adjusted R²** | 0.8958 | | | | | | |
|  | **AICc** | 444.5985 | | | | | | |
| **Stroke** | **Mean** | 0.1994 | 0.5681 | 0.1363 | -0.2162 | -0.1594 | 0.3133 | 0.6839 |
|  | **Median** | 0.1985 | 0.5302 | 0.0196 | -0.2156 | -0.1294 | 0.3828 | 0.8013 |
|  | **SD** | <0.0016 | 0.2146 | 0.1299 | 0.024 | 0.128 | 0.2046 | 0.3051 |
|  | **Adjusted R²** | 0.7974 | | | | | | |
|  | **AICc** | 969.7137 | | | | | | |

Residual Diagnostics of OLS Models

To evaluate the spatial performance and model fit of the OLS regressions, standardized residuals were mapped for each health outcome. Standardized residuals indicate the deviation of observed values from predicted values in standard deviation units, allowing for identification of areas where the model consistently over- or under-predicts. Spatial clustering or large positive/negative residuals may suggest the presence of spatial nonstationarity or missing explanatory variables. Visual inspection of the residual maps can help assess model assumptions and reveal patterns of local model misspecification. These residual diagnostics complement global fit statistics such as Adjusted R² and AICc by highlighting localized discrepancies in model performance.

Figure S1. Standardized Residuals from Ordinary Least Squares (OLS) Regression for Blood Pressure

Figure S2. Standardized Residuals from Ordinary Least Squares (OLS) Regression for Cancer

Figure S3. Standardized Residuals from Ordinary Least Squares (OLS) Regression for COPD

Figure S4. Standardized Residuals from Ordinary Least Squares (OLS) Regression for Asthma

Figure S5. Standardized Residuals from Ordinary Least Squares (OLS) Regression for Mental Health

Figure S6. Standardized Residuals from Ordinary Least Squares (OLS) Regression for Diabetes

Figure S7. Standardized Residuals from Ordinary Least Squares (OLS) Regression for Stroke
